# OH(^2^Π) + C_2_H_4_ Reaction: A Combined Crossed Molecular Beam and Theoretical Study

**DOI:** 10.1021/acs.jpca.2c08662

**Published:** 2023-05-19

**Authors:** Pengxiao Liang, Emília Valença Ferreira de Aragão, Lisa Giani, Luca Mancini, Giacomo Pannacci, Demian Marchione, Gianmarco Vanuzzo, Noelia Faginas-Lago, Marzio Rosi, Dimitrios Skouteris, Piergiorgio Casavecchia, Nadia Balucani

**Affiliations:** † Dipartimento di Chimica, Biologia e Biotecnologie, Università Degli Studi di Perugia, Perugia 06123, Italy; ‡ Master-Tec Srl, Via Sicilia, 41, Perugia 06128, Italy; § Université Grenoble Alpes, 621 Av. Centrale, Saint-Martin-d’Hères 38400, France; ∥ Dipartimento di Ingegneria Civile Ed Ambientale, Università Degli Studi di Perugia, Perugia 06125, Italy

## Abstract

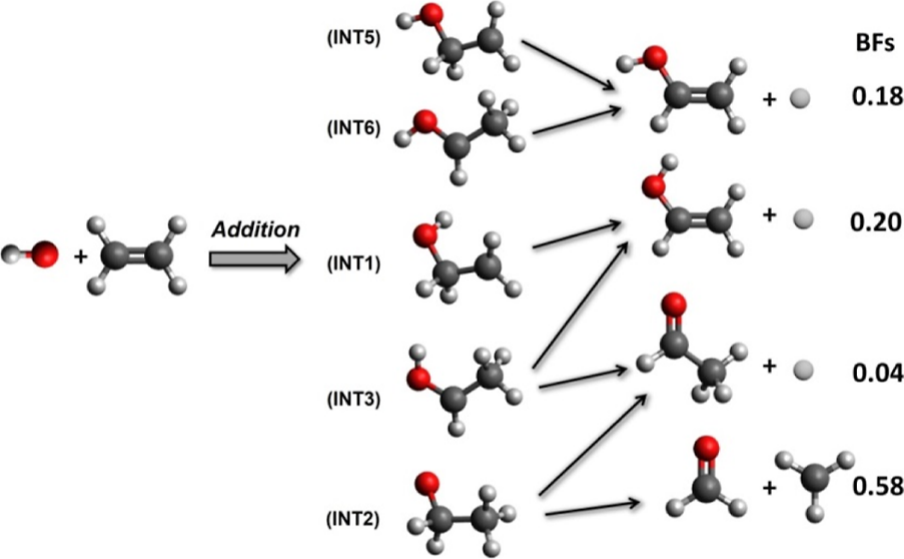

The reaction between the ground-state hydroxyl radical, OH(^2^Π), and ethylene, C_2_H_4_, has been investigated under single-collision conditions by the crossed molecular beam scattering technique with mass-spectrometric detection and time-of-flight analysis at the collision energy of 50.4 kJ/mol. Electronic structure calculations of the underlying potential energy surface (PES) and statistical Rice–Ramsperger–Kassel–Marcus (RRKM) calculations of product branching fractions on the derived PES for the addition pathway have been performed. The theoretical results indicate a temperature-dependent competition between the *anti*-/*syn*-CH_2_CHOH (vinyl alcohol) + H, CH_3_CHO (acetaldehyde) + H, and H_2_CO (formaldehyde) + CH_3_ product channels. The yield of the H-abstraction channel could not be quantified with the employed methods. The RRKM results predict that under our experimental conditions, the *anti*- and *syn*-CH_2_CHOH + H product channels account for 38% (in similar amounts) of the addition mechanism yield, the H_2_CO + CH_3_ channel for ∼58%, while the CH_3_CHO + H channel is formed in negligible amount (<4%). The implications for combustion and astrochemical environments are discussed.

## 1 Introduction

Besides being of fundamental interest, the reaction of the hydroxyl radical (OH) with the ethylene (C_2_H_4_) molecule is of great relevance in combustion chemistry^1^ and atmosphere chemistry,^2^ as it is considered one of the first steps in the oxidation of ethylene. It also plays a role in the chemistry of interstellar medium (ISM),^3,4^ where both OH^5−12^ and C_2_H_4_^13,14^ and their possible products, acetaldehyde^15−24^ and vinyl alcohol,^4,25,26^ have been observed. Given its importance, it has been widely investigated over the past several decades from both experimental (mostly kinetic) and theoretical points of view. In contrast, little is known about its reaction dynamics; in particular, the dynamics of the OH + C_2_H_4_ bimolecular reaction has never been investigated experimentally under single-collision conditions.

Kinetic experiments have been performed to obtain information on the rate coefficients and their dependence on temperature and pressure,^27−40^ and kinetic and photochemical investigations to derive product branching fractions have also been reported.^41−46^ Additionally, theoretical calculations have been carried out to derive the relevant potential energy surface (PES) and to obtain a theoretical estimate of the rate coefficients through high-level ab initio electronic structure and kinetic (statistical) calculations.^47−59^ According to these studies, the OH + C_2_H_4_ reaction can proceed through two different mechanisms, that is, either by the H-abstraction mechanism, leading to the formation of a water molecule and the vinyl radical (C_2_H_3_), or by the addition mechanism of the OH radical to the C=C double bond of ethylene. While the direct abstraction process exhibits a sizeable entrance energy barrier, the addition process appears to be barrierless and is characterized by the initial formation of a pre-reactive van der Waals complex, which leads to the formation of the C_2_H_4_OH (2-hydroxyethyl) covalently bound radical intermediate through a submerged barrier. Further evolution of the initial addition intermediate has been a central issue for the detailed characterization of the kinetics and dynamics of this reaction.^52^

The relevant possible product channels of the elementary OH + C_2_H_4_ reaction from low temperature (*T*) (96–298 K) up to high *T* (∼1100–2500 K), according to a detailed theoretical work,^52^ are the following:

Addition mechanism
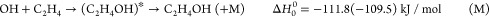

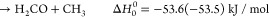
1
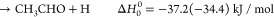
2
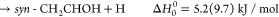
3
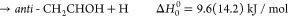
4

Abstraction mechanism
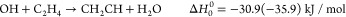
5

The enthalpies of reaction are from literature,^40,60−62^ with values in parenthesis being the theoretical values from the electronic structure calculations of the present work at the CCSD(T)/aug-cc-pVTZ level of theory (Section 2.2).

To put our work in right context, we will briefly summarize a few key investigations among the many available on this system, covering (i) its reaction kinetics as a function of temperature (*T*) and pressure (*p*),^27−40^ (ii) the theoretical calculations of the underlying PES and related kinetic (statistical) computations of channel-specific rate constants and product branching fractions (BFs) as a function of temperature and pressure,^50,52^ and (iii) the experimental product detections and BFs estimated starting from the OH and C_2_H_4_ reactants at 295 K and 2 Torr in a flow system,^41^ and from the chemically activated C_2_H_4_OH intermediate (2-hydroxyethyl radical) produced by laser-photodissociation of 2-bromoethanol at 202–215^42^ and at 193 nm.^43−45^

### 1.1 Kinetic Studies

The kinetics of the title reaction has been extensively studied since the early 1970s.^27−40,63−67^ The experimental data at temperatures lower than 600 K are consistent with a reaction mechanism which proceeds via OH addition to the π bond of ethylene and subsequent collisional stabilization of the energy rich C_2_H_4_OH adduct [channel (M)]. The observation that the reaction rate decreases as *T* rises indicates that the addition reaction is barrierless, forming initially a van der Waals complex (C_2_H_4_···OH) that will eventually evolve, via a submerged barrier, to a covalently bound C_2_H_4_OH radical intermediate (2-hydroxyethyl). This initial intermediate can (i) dissociate back to reactants, (ii) loose an H atom, or (iii) isomerize by hydrogen shift to other intermediates: CH_3_CH_2_O (ethoxy radical) and CH_3_CHOH (1-hydroxyethyl radical), which in turn can emit a CH_3_ radical [channel (1)] and an H atom [channels (2–4), respectively]. Notably, at *T* > 700 K, the reaction rate starts increasing, and this is due to a mechanism change attributed mainly to the onset of the direct abstraction mechanism forming H_2_O [channel (5)]. The observed large deviations from Arrhenius behavior of the rate constant as a function of *T*, featuring a dramatic change in activation energies around 800 K (*E*_a_ ∼ −4 kJ/mol below 560 K, while *E*_a_ ∼ 16–21 kJ/mol above 720 K),^33^ indicate clearly a switching of the dominant reaction mechanism. In particular, as *T* rises, the probability of stabilization of the initial C_2_H_4_OH radical intermediate decreases while its isomerization processes increase in importance, and at high *T*, the direct H abstraction by OH tends to become dominant.

Experimentally, rate coefficients have been measured down to 96 K^37^ and up to shock-tube (1100–1300 K)^63−66^ and flame (2150 K)^67^ temperatures. These studies have permitted us to investigate the competition between (i) the addition-stabilization [channel (M)], (ii) the addition-isomerization followed by dissociation to bimolecular products [channels (1), (2), (3), and (4)], and (iii) the direct abstraction [channel (5)]. It is accepted that at low temperatures the reaction proceeds completely by addition and (collisional) stabilization of the first addition radical intermediate; however, as *T* increases, stabilization becomes less efficient, and at high *T*s, the possible isomerization channels that can lead to H [channels (2), (3), and (4)] and also CH_3_ elimination [channel (1)], and the H-abstraction [channel (5)] dominate the reaction mechanism. The formation of these bimolecular channels involves rather high isomerization barriers, some of which are located above the energy of the reactants.

It is certainly very valuable to explore the competition between the above possible channels as a function of *T*. This can be done more readily theoretically (Section 1.2), while experimentally it is quite challenging; yet, a few experimental studies on the determination of product BFs in different energetic conditions are available, both in a flow system (Section 1.3)^41^ and in photochemical experiments in a molecular beam (Section 1.5).^42−45^

### 1.2 Theoretical Studies

The kinetics of the association reaction to C_2_H_4_OH stabilization [channel (M)] has been investigated quite extensively also from a theoretical perspective.^47−59^ Notably, there has been substantial disagreement among high *T* (1200–1300 K) kinetic measurements and between extrapolation of these data and shock tube experiments at higher *T* (1850–2150 K) (see^52^). Furthermore, results from theoretical studies of the abstraction pathway, which is dominant at high T, differed substantially among them (see^52^). In 2006, Senosiain et al.^52^ reexamined theoretically the OH + C_2_H_4_ reaction with the aim of bridging the existing (at that time) experimental data from low- and high-temperature kinetic measurements using a sound theoretical model. They developed a high-level PES and used the model of Greenwald et al.^48^ in conjunction with a multichannel-master equation treatment. They predicted channel-specific rate coefficients as a function of *T* (from 250 up to 2500 K) and *p* (from the collisionless limit up to 100 bar). The theoretical results confirmed the importance (BF ∼90%) of the H abstraction channel (5) at *T* > 800 K (at *p* ∼ 1 atm of N_2_). In the collisionless limit (see Figure 11 in^52^), the BF of channel (5) was found to be nearly constant (∼90%) from 2500 down to ∼400 K, while it was found decreasing somewhat as *T* decreases further, with a value of ∼70% at 250 K. At *T* > 800 K (up to 2500 K), it was found that a significant fraction (BF ∼ 10%) of the reaction leads to vinyl alcohol (ethenol) + H [the sum of channels (3) and (4)], and flame calculations based on the predicted rate coefficients were found to account successfully for the amount of vinyl alcohol observed in flames by Taatjes et al.^68^ Notably, in the collisionless limit, the vinyl alcohol forming channel was predicted to decrease to nearly zero at *T* ≤ 300 K, while the H_2_CO + CH_3_ channel (1) was predicted to be substantial (∼10% at 300 K), then decreasing rapidly to ∼0.7% at 1000 K, and even lower at higher *T*. In contrast, the CH_3_CHO + H channel (2) was found to be less than 1% over the entire range 300–2500 K.^52^

### 1.3 Product Branching Fractions in the Flow System

It is worth noting that the early experimental study by Bartels et al.^41^ at low temperatures (295 K) and low pressures (2 Torr) (in He bath gas) using discharge flow reactors and a Laval nozzle reactor and quantitative product analysis by mass spectrometry with molecular beam sampling determined that the products of the OH + C_2_H_4_ reaction consist of 21% stabilized C_2_H_4_OH addition intermediate [channel (M)], 44% H_2_CO + CH_3_ [channel (1)], and 35% C_2_H_4_O (acetaldehyde) + H [channel (2)]. In particular, the abstraction reaction [channel (5)] was found to be negligible (BF < 2.5%). Clearly, the addition intermediate was stabilized by multiple collisions in the above study.

It should be noted that the theoretical work of Senosian et al.^52^ did not examine these previous experimental results of 1982,^41^ and therefore no theoretical predictions at 295 K and 2 Torr (in He bath gas) were made. However, the theoretical BFs of Senosian et al.^52^ could well be in line with those early kinetic results. In fact, the calculations at 300 K and 7.6 Torr (with N_2_ bath gas) predict about 0.5% abstraction, 0.06% of CH_3_ + H_2_CO, 0.003% CH_3_CHO + H, and 0.001% CH_2_CHOH + H, with 99.4% being stabilized C_2_H_4_OH. However, at 2 Torr (with He bath gas), stabilization is expected to decrease substantially with respect to 7.6 Torr, and being abstraction relatively unimportant at 300 K (only 0.5% at 7.6 Torr and N_2_ bath gas), the yields of the CH_3_ and H forming channels could well make CH_3_ and H formation the dominant channels, as observed by Bartels et al.^41^

### 1.4 Flame Studies with Synchrotron Radiation

More than 20 years after the early study of Bartels et al.,^41^ flame studies by Taatjes et al.^52,68^ observed vinyl alcohol formation from the OH + C_2_H_4_ reaction, and this observation triggered most of the recent renewed interest in the kinetics and dynamics of this reaction. These studies^52,68^ suggested that the vinyl alcohol forming channel should be certainly included in hydrocarbon oxidation mechanisms.

### 1.5 Photochemical Studies

A few years after the theoretical work of Senosiain et al.,^52^ two nearly simultaneous experimental investigations were carried out with the purpose of exploring the critical regions of the PES for product branching from the addition reaction. Reisler and co-workers^42^ observed (by 1 + 1′ REMPI) the formation of D atoms from the predissociation of rovibrationally excited, partially deuterated 2-hydroxyethyl radicals, CD_2_CD_2_OH, produced from the photolysis of 2-bromoethanol at 202–215 nm in a molecular beam. The *D* time-of-flight (TOF) distributions could be associated with vinyl alcohol and/or acetaldehyde coproducts. The possible *D* coproducts were also characterized by theory. From the measured TOF distributions and RRKM calculations based on the PES of Senosiain et al.,^52^ the authors concluded that, under the experimental conditions, the predominant (∼99%) dissociation channel of the CD_2_CD_2_OH radical is OH + C_2_D_4_, and only about 1% leads to D formation. It was further concluded that the vinyl alcohol forming channel is strongly favored over the acetaldehyde-forming channel at all excess energies (about 60–95 kJ/mol with respect to the reagent asymptote of the OH + C_2_D_4_ reaction). At about the same time, Butler and co-workers^44^ measured (by ion imaging techniques with 10.5 eV laser photoionization detection) the velocity distribution of the stable radicals arising from the dissociation of rovibrationally excited 2-hydroxyethyl radicals generated from the photodissociation of 2-bromoethanol at 193 nm. They analyzed the product distribution from the predissociation of chemically activated C_2_H_4_OH having an excess energy, above the OH + C_2_H_4_ reactant asymptote, of ∼110–160 kJ/mol, a value somewhat larger than in the study of Reisler and co-workers^42^ because of the shorter photolysis wavelength used (193 nm vs 202–215 nm). The energy of the transition states calculated by Senosiain et al.^52^ was used also in this study to derive the product BFs. Again, similarly as in the study at longer wavelengths, the RRKM rate constants predicted more than 99% of the C_2_H_4_OH radical dissociating to OH + C_2_H_4_. Of the small reactive portion, the BF of H + vinyl alcohol was determined to be 58%, that of H_2_CO + CH_3_ 39%, and that of H + acetaldehyde 3%. These results for the H-forming channels are in line with those by Reisler and co-workers,^42^ with the substantial addition that also the methyl-forming channel was quantified. Shortly afterward, Butler and co-workers^45^ performed a similar but improved experiment at the same photolysis wavelength (193 nm), exploiting mass-spectrometric detection with tunable vacuum ultraviolet synchrotron radiation (soft ionization) and TOF analysis. In this more detailed study, the authors were able to probe all possible dissociation products (product asymptotes) of the rovibrationally excited CH_2_CH_2_OH radical intermediate: OH + C_2_H_4_, H_2_O + C_2_H_3_, CH_2_CHOH + H, H_2_CO + CH_3_, and CH_3_CHO + H, for which the following branching fractions were derived: 0.765:0.145:0.026:0.063:<0.01, respectively. Notably, the relative ratio vinyl alcohol/acetaldehyde/formaldehyde of 0.29:0.001:0.71 obtained in the new experiment^45^ differed somewhat from the previous determinations (0.58:0.03:0.39)^44^ using ion imaging detection with 10.5 laser photoionization. The acetaldehyde channel was minor in both cases.

It should be remarked that both photochemical studies^44,45^ found the H + vinyl alcohol channel to be strongly favored with respect to the H + acetaldehyde channel, in agreement with the theoretical predictions. Interestingly, the studies of Butler and co-workers found the yield of the H channels comparable to the CH_3_-forming channel (61% vs 39%)^44^ and (36% vs 64%).^45^ which is similar to what was found by Bartels et al.,^40^ in the flow study at 295 K and 2 Torr. We note, however, that according to the statistical predictions of Senosiain et al.^52^ the yields of these H and CH_3_-forming competing channels are comparable at 600 K, and the ratio H/CH_3_ becomes larger than unity at *T* ≥ 700 K (see Figure 11 in^52^).

### 1.6 Present Work

Despite the above detailed investigations and all the work done, a study of the real title bimolecular reaction in dynamics experiments is still missing. In the present paper, we report the first dynamical investigation of the OH + C_2_H_4_ bimolecular reaction using the crossed molecular beam (CMB) scattering method with mass-spectrometric detection and TOF analysis at the collision energy, *E*_c_, of 50.4 kJ/mol. Under our experimental conditions, we were able to explore experimentally only the dynamics of the H displacement processes following OH addition to ethylene. We could neither explore the abstraction dynamics because of experimental complications nor the dynamics of the H_2_CO + CH_3_ channel because the latter is kinematically very unfavorable with respect to the H-forming channels for which the reactive scattering signal is already very small. The experimental work has been assisted by dedicated electronic structure calculations of the PES for both addition and abstraction mechanisms, and statistical computations of the BFs of all possible products arising from the addition mechanism performed under the conditions of our CMB experiments and also in other conditions for comparison with previous data. Our combined experimental/theoretical approach confirms that, under single-collision conditions, the dominant H-displacement channel is the one leading to vinyl alcohol rather than acetaldehyde. This is in line with previous conclusions from photodissociation experiments on 2-bromoethanol^42,44,45^ as well as from theoretical studies.^52^ In addition, the pathways to the two conformers, *anti-* and *syn-*vinyl alcohol, and their relative BF have been theoretically distinguished since they have different spectroscopical features. Indeed, one of the aims of the present study was to verify whether the title reaction is a viable formation route of acetaldehyde and/or *anti-* and *syn-*vinyl alcohol in warm or hot regions of the ISM. In the latter regard, the present investigation falls within the EC project “Astro-Chemical Origins” (ACO).

The paper is organized as follows: Section 2 describes the experimental and theoretical methods. The experimental results are presented in Section 3, while the theoretical ones are in Section 4. Discussion follows in Section 5. The astrochemical implications are discussed in Section 6. Finally, conclusions are given in Section 7.

## 2 Experimental and Theoretical Methods

### 2.1 Experimental Method

The CMB apparatus used in the present experiment has been described in detail elsewhere,^69−75^ and only a brief description is given here. Two supersonic beams of the reactants were crossed at the angle of 90° in a large scattering chamber kept, by extensive turbo- and cryo-pumping, at about 7 × 10^–7^ mbar under operation to ensure single-collision conditions. The scattered species were recorded by a triply differentially pumped ultra-high-vacuum detector equipped with a tunable electron impact ionizer, followed by a quadrupole mass filter and a Daly-type ion detector. The whole detector unit can be rotated in the collision plane, around the axis orthogonal to the scattering plane and passing through the intersection of the two reactant beams. The angular distribution of products, *N*(Θ), was measured as a function of the laboratory scattering angle Θ (Θ = 0° corresponds to the OH beam direction). The noise was subtracted by modulating the molecular beam with a turning fork chopper at a frequency of 160 Hz. The velocity distribution of reactants and products was derived from the corresponding TOF distribution, *N*(Θ, *t*), by using the single-shot and cross-correlation method,^76^ respectively.

The OH radical beam was generated from a radio-frequency (RF) discharge beam source.^77^ Helium gas was bubbled into a glass vial containing H_2_^18^O maintained at 277 K. By discharging 160 mbar of He/H_2_^18^O gas mixture at 300 W RF power and expanding it through a water-cooled quartz nozzle with a 0.24 mm diameter, a supersonic beam of ^18^OH radical with a peak velocity of 2866 m/s and a speed ratio of 4.5 was produced. Beside ^18^OH radicals, the beam also contains other species, such as H atoms, ^18^O(^3^P,^1^D) atoms, as well as undissociated H_2_^18^O molecular precursor. In particular, the presence of ^18^O atoms could represent a complication due to their high reactivity with C_2_H_4_.^73^ However, the signal at the mass-to-charge ratio, *m*/*z*, of 46 corresponding to the parent peak of products with the gross formula C_2_H_3_^18^OH can only be originated by the reaction between ^18^OH and C_2_H_4_. The ^18^OH radicals are in the ground ^2^Π electronic state because the radiative lifetime of possible electronically excited states of OH is less than 1 μs,^78^ which is much shorter than the time needed by the ^18^OH radicals in the beam to reach the collision center (about 5 cm away from the nozzle).^78,79^ According to previous characterizations of the internal state population by the laser-induced fluorescence (LIF) technique, the ^18^OH radicals are in the lowest several rotational levels and in the ground vibrational level (>99%).^78^ The supersonic beam of ethylene was generated by expanding the pure gas at the stagnation pressure of 800 mbar through a 0.1 mm diameter stainless-steel nozzle held at room temperature. The peak velocity of C_2_H_4_ was 825 m/s with a speed ratio of 6.0. The resulting collision energy, *E*_c_, of the experiment was 50.4 kJ/mol, while the center-of-mass position angle, Θ_CM_, was 23.7°.

To gain quantitative and physically meaningful information on the chemical dynamics, it was necessary to convert our observables from the laboratory (LAB) reference frame to the privileged center-of-mass (CM) reference frame. This has been achieved by a forward convolution program where trial CM product angular, *T*(θ), and translational energy, *P*(*E*_T_′), distributions for the detected channel are given as an input to derive the LAB and TOF distributions. In this approach, the reactive differential cross section is assumed to be separable into two independent functions, that is I_CM_(θ, *E*_T_′) = *T*(θ) × *P*(*E*_T_′). The angular distributions and TOF spectra in the LAB frame are calculated using trial CM *T*(θ) and *P*(*E*_T_′) distributions, and the procedure is repeated until the best-fit of the experimental data is achieved.^70,74^

### 2.2 Electronic Structure Calculations of the PES

The PES for the OH(^2^Π) + C_2_H_4_ system was investigated through the optimization of the most stable stationary points along the reactive channels. Using a well-established computational scheme described in previous works from our group,^80−86^ the geometries of minima and saddle points were optimized with a cost-effective method, and single-point calculations were then performed employing a more expensive method to obtain accurate energies. More specifically, geometry optimization calculations and vibrational frequency analysis were performed using density functional theory with the Becke-3-parameter exchange and Lee–Yang–Parr correlation (B3LYP)^87,88^ in conjunction with the correlation consistent valence polarized basis set aug-cc-pVTZ.^89^ Vibrational frequency analysis was performed in order to obtain the energy correction at 0 K and to determine the nature of each stationary point, namely a minimum if all frequencies are real and a saddle point if there is one and only one imaginary frequency. Intrinsic reaction coordinate (IRC) calculations^90,91^ were performed for each saddle point geometry in order to correctly assign each transition state on the PES. Finally, the energies of the stationary points were computed with the more energetically accurate coupled-cluster CCSD(T) method.^92−94^ The energies presented throughout the paper correspond to the CCSD(T) energies corrected with zero-point energies from the frequency calculations. All calculations were performed adopting an unrestricted formalism using the Gaussian 09 code,^95^ while the analysis of the vibrational frequencies was performed using the Avogadro software.^96^

### 2.3 Kinetic Calculations

The kinetics of the neutral–neutral reaction OH(^2^Π) + C_2_H_4_ was investigated using a code implemented in our group for this purpose.^97−99^ In details, following the RRKM scheme,^100^ the microcanonical rate constant *k*(*E*) for a specific reaction at a specific total energy is given by the expression
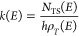
6where *N*_TS_(*E*) represents the sum of states of the transition state at energy *E*, ρ(*E*) is the density of states of the reactants, and *h* is Planck’s constant. The partition function was used to perform an inverse Laplace transform in order to evaluate the rotational densities of states both for the reactants and for the transition states. Subsequently, the rotational densities of states were convoluted with the corresponding vibrational ones using a direct count algorithm. Finally, the sum of states *N*(*E*) was obtained by integrating the relevant density of states up to energy *E*, and the rigid rotor/harmonic oscillator model was assumed. Where possible, tunneling (as well as quantum reflection) was considered by using the corresponding imaginary frequency of the transition state and calculating the tunneling probability for the corresponding Eckart barrier. The rate of the inverse capture step (back dissociation) was calculated considering the densities of states of the reactants and the initial intermediate using a detailed balance argument. For the cases in which we were not able to locate a clear transition state in the exit channel, the corresponding microcanonical rate constant was obtained through a variational approach: *k*(*E*) was evaluated at various points along the reaction coordinate, and the point which minimizes the rate constant was chosen in accordance with the variational transition state theory.^101^ After the calculation of all microcanonical rate constants, a Markov (stochastic) matrix was setup for all intermediates and final channels to derive the product branching fractions for the overall reaction. *k*(*E*) was subsequently Boltzmann averaged for each temperature of interest to yield *k*(*T*).

## 3 Experimental Results

**Figure 1:**
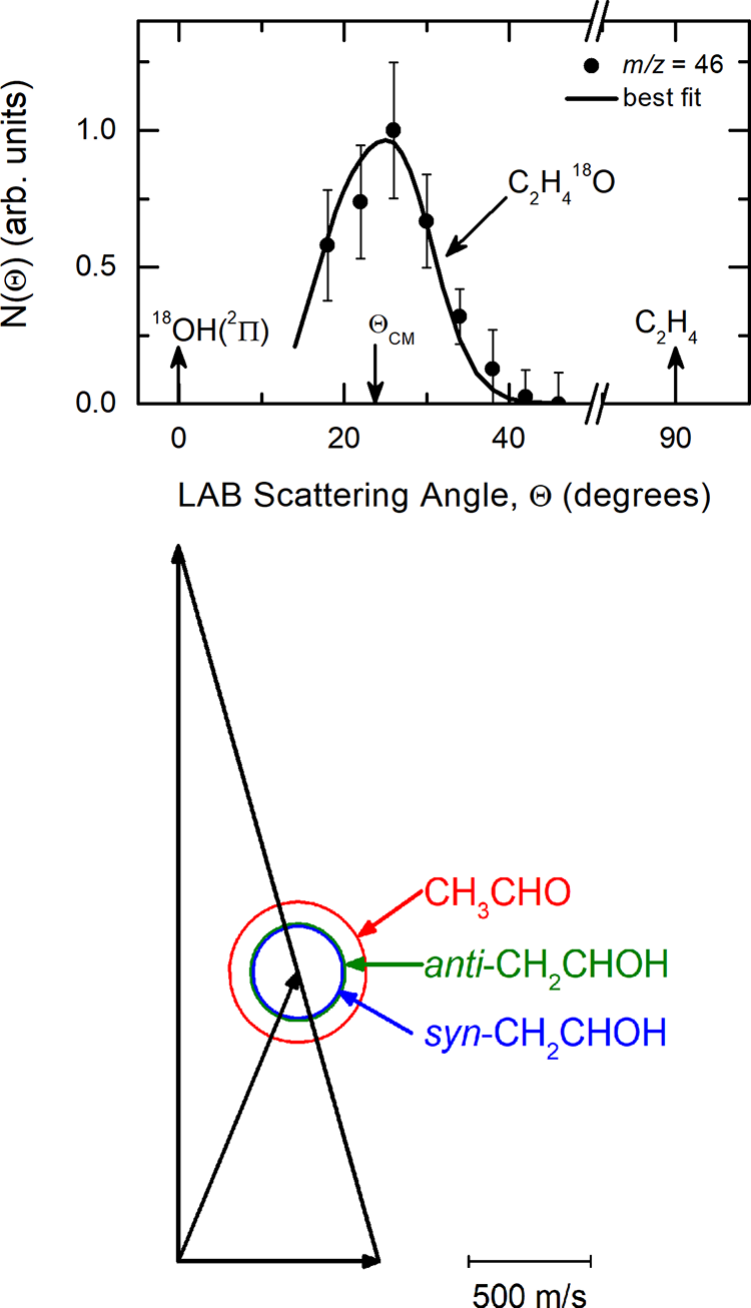
Top: LAB angular distribution collected at *m*/*z* = 46 (C_2_H_4_^18^O^+^) for the ^18^OH (^2^Π) + C_2_H_4_ reaction. At *E*_c_ = 50.4 kJ/mol. The filled circles are the normalized experimental data with indicated error bars (±1σ), while the black line is the calculated distribution when using the best-fit CM functions displayed in Figure 3. Θ_CM_ indicates the center-of-mass angle. Bottom: Velocity vector (Newton) diagram showing the circles that delimit the angular range and center-of-mass speed for the products of the H-elimination channels (2), (3), and (4). The circles delimit the maximum velocity that the three possible isomers with the gross formula C_2_H_4_^18^O can attain if all the available energy is channeled into product translational energy. Red continuous line: Newton circle for the CH_3_CH^18^O product; Blue continuous line: Newton circle for the *syn*-CH_2_CH^18^OH product; Olive continuous line: Newton circle for the *anti*-CH_2_CH^18^OH product.

**Figure 2:**
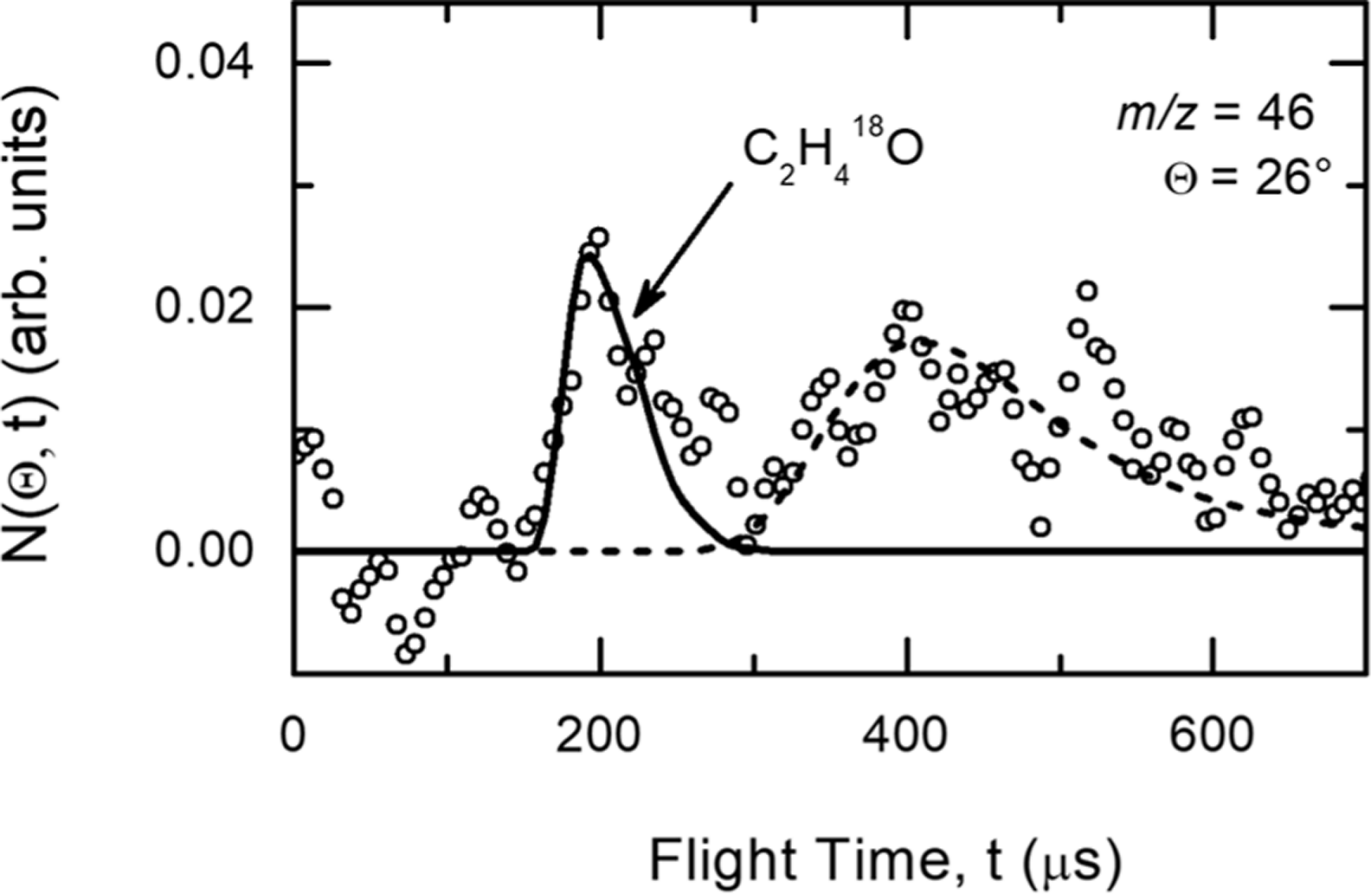
LAB time-of-flight spectrum recorded at *m*/*z* = 46 (C_2_H_4_^18^O^+^) from the ^18^OH(^2^Π) + C_2_H_4_ reaction. The open circles indicate the experimental data, while the black line is the calculated distribution when using the best-fit CM functions displayed in Figure 3. The slow feature peaking around 400 μs is a thermal component of the C_2_H_3_^18^OH product arising from the effusive (thermalized) components of the two reactant beams (see text).

Reactive scattering signal was recorded at *m*/*z* = 46, corresponding to the parent ion of the heavy coproducts (with a gross formula of C_2_H_4_^18^O) of the H-displacement channels. Isotopically labeled ^18^OH radicals were used to prevent the interference from high CO_2_ background gas at *m*/*z* = 44. The recorded LAB angular distribution, together with the Newton diagram illustrating the kinematics of the experiment, are shown in Figure 1 (top and bottom panels, respectively). The relatively large error bars (representing ±1σ of the average of five angular scans with counting times of 100 s at each angle) are due to the low signal-to-noise (S/N) ratio of the experiment. Because of the very low reactive signal, only one TOF spectrum could be recorded (counting time about 4 h) at Θ = 26° (about 2° away from Θ_CM_) and is displayed in Figure 2. The relatively narrow TOF peak at ∼200 μs is from the C_2_H_4_^18^O reactive scattering signal from the ^18^OH + C_2_H_4_ reaction, while the slower, broader component, peaking near 400 μs, is associated with some source of thermal signal, possibly caused by the reaction of the effusive parts (thermalized at about 300 K) of the two reactant beams. This has been modeled assuming a slightly polarized *T*(θ) and a *P*(*E*_T_′) somewhat slower than the best-fit *P*(*E*_T_′).

We were unable to record the reactive signal associated to the H_2_CO + CH_3_ channel (1) because of a strong interfering signal coming from the reaction of atomic oxygen (also present in the OH beam) with ethylene that affected both *m*/*z* = 30 (H_2_CO^+^) and *m*/*z* = 15 (CH_3_^+^). We could neither probe the H_2_^18^O product nor the C_2_H_3_ coproduct from the abstraction channel (5) because of strong elastic interferences from the ^18^O beam, which contains also undissociated H_2_^18^O, and from the C_2_H_4_ beam because of the strong dissociative ionization of ethylene at *m*/*z* = 27 (C_2_H_3_^+^).

**Figure 3:**
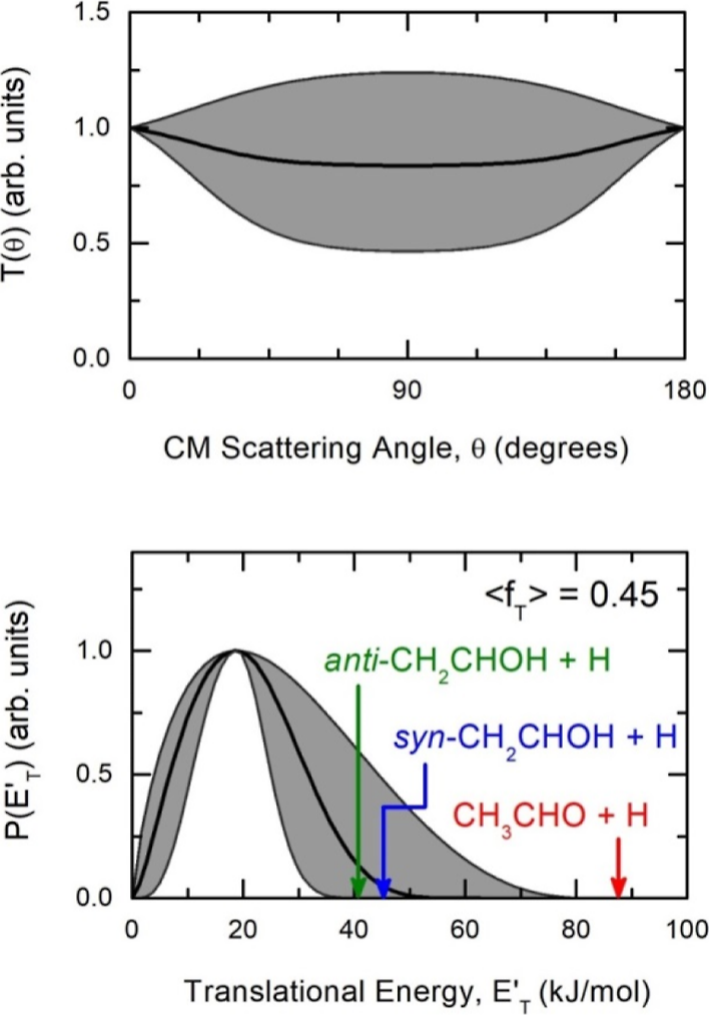
Best-fit CM product angular distribution (top-panel) and translational energy distribution (bottom-panel). The solid black lines indicate the best-fit function. The shaded areas delimit the range of functions which still provide an acceptable fit of the LAB data. The arrows in the bottom-panel indicate the maximum available energy for the three different isomeric product channels by taking into account the energy conservation principle (*E*_T*O*T_ = *E*_c_ – Δ*H*^0^). The value of ⟨*f*_T_⟩ is the fraction of the total available energy released as product translational energy when considering the second most exothermic H-elimination product channel, i.e., *syn*-CH_2_CHOH + H [channel (3)].

The LAB angular distribution of Figure 1 and the TOF spectrum of Figure 2 could be fit by using one set of CM functions. Figure 3 shows the best-fit CM angular and translational energy distributions for the H-displacement channels from the reaction of ^18^OH with C_2_H_4_ forming C_2_H_4_^18^O products. The shaded areas in Figure 3 delimit the range of CM functions that still provide an acceptable fit of the experimental data. Within the uncertainty of the present determination, the maximum value of *E*_T_′ is compatible with the energetics of any of the three possible products with mass 46, i.e., CH_3_CH^18^O, *syn*-CH_2_CH^18^OH, and *anti*-CH_2_CH^18^OH. The best-fit CM *T*(θ) is distributed over the entire 0–180° CM angular range and is forward-backward symmetric, nearly isotropic, but exhibits error bounds that are consistent with both polarization and preference for sideways scattering (*T*(90°)/*T*(0°) as high as 1.24). These features imply that the reaction mechanism proceeds via the formation of a long-lived C_2_H_5_^18^O complex,^102−104^ which fragments via atomic hydrogen loss to form the C_2_H_4_^18^O product(s). The best-fit translational energy distribution, *P*(*E*_T_′), is characterized by a relatively fast rising edge with a maximum peak value away from zero, located around 18–20 kJ/mol. This value suggests that the exit barrier is likely tight. The average translational energy, <*E*_T_^′^>, of the C_2_H_4_^18^O + H product(s) (where < *E*_T_^′^> = ) was derived to be ∼18 kJ/mol, indicating that the average fraction, <*f*_T_>, of the maximum total available energy, *E*_TOT_ (where *E*_TOT_ = *E*_c_ – Δ*H*_0_^0^) released into the translational degrees of freedom of the products is 0.45 (assuming *E*_TOT_ of *syn*-CH_2_CHOH). We remind that < *f*_T_> is defined as <*f*_T_> = <*E*_T_^′^>/*E*_TOT_.

The best-fit of both angular and TOF distributions is only slightly affected by the high-energy cutoff of the *P*(*E*_T_′), while it is more sensitive to the rising edge and peak position. A good fit of the experimental distribution can be achieved with a cutoff of about 50_–15_^+25^ kJ/mol. This cutoff value appears consistent with the total available energy for channels (3) and (4), but a minor contribution from also channel (2) cannot be excluded. To elucidate the detailed reaction mechanisms under the present experimental conditions and separate the relative BFs of channels (2), (3), and (4), calculations of the PES and BF statistical calculations are pivotal to assist the experimental data analysis.

## 4 Theoretical Results

### 4.1 Electronic Structure Calculations

**Figure 4:**
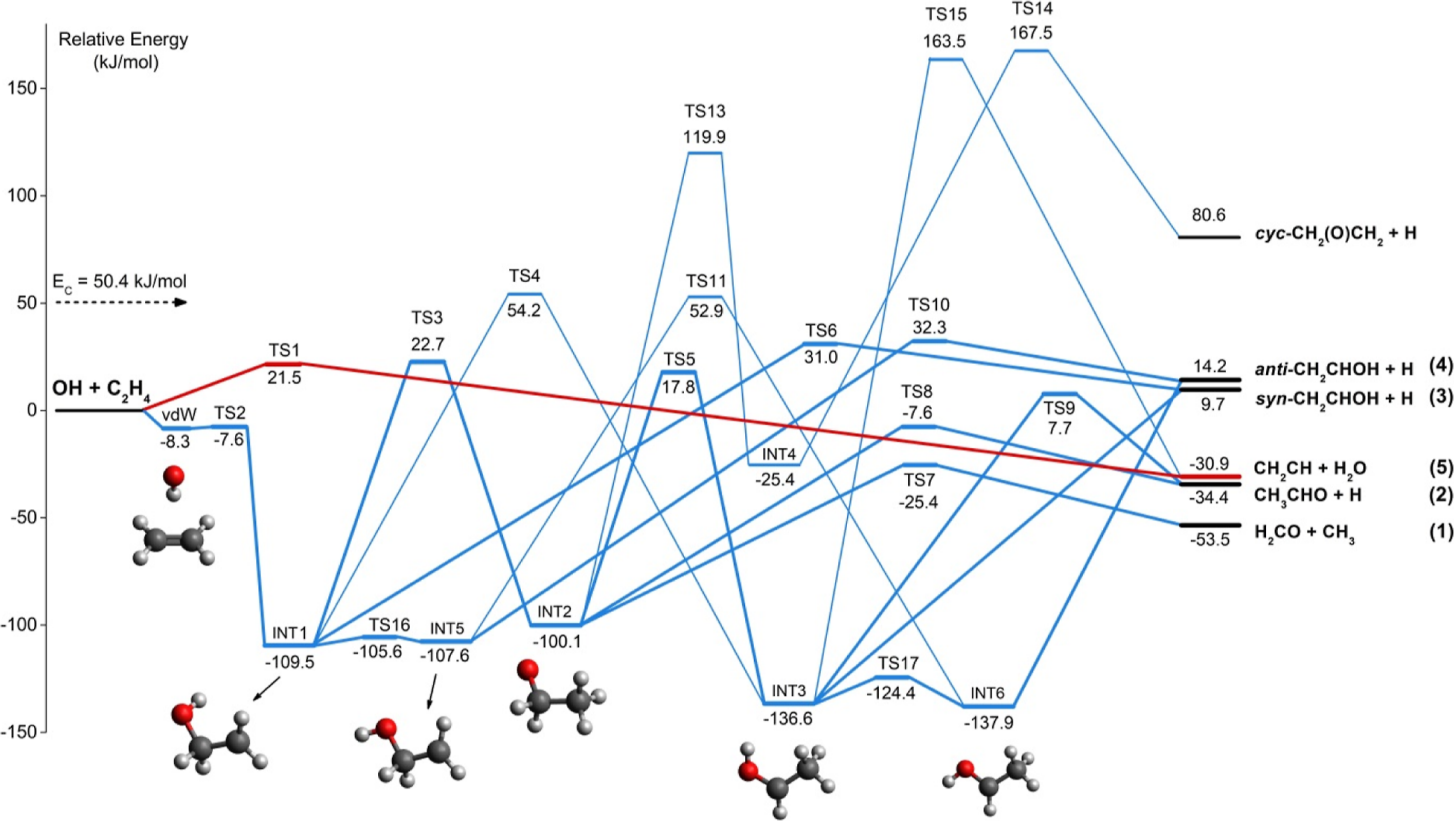
Schematic representation of the global PES for the system OH(^2^Π) + C_2_H_4_ with energies calculated at the CCSD(T)/aug-cc-pVTZ level of theory.

A schematic representation of the C_2_H_5_O PES obtained for the OH(^2^Π) + C_2_H_4_ reaction is reported in Figure 4. All the reported energy values, computed at the CCSD(T)/aug-cc-pVTZ//B3LYP/aug-cc-pVTZ level of theory, are expressed with respect to the reactant energy asymptote, considered as zero energy. As a complement, Table S1 in the Supporting Information lists the energy of all unimolecular processes involved in the PES, while a comparison of the values of energy barriers calculated in this work with those determined in previous theoretical studies at different levels of theory is reported in Table S2 of the Supporting Information. The optimized geometry of all the stationary points of the PES, with distances in Angstroms and bond angles in degrees, is reported in Figures S1–S3 of the Supporting Information. For completeness in the Supporting Information, we also include the cartesian coordinates for all optimized geometries and the frequencies (Table S3).

In our PES, we have distinguished the rotamers INT1/INT5 and INT3/INT6. This is essential to evaluate the branching fractions of *anti*-CH_2_CHOH and *syn*-CH_2_CHOH. INT1/INT5 and INT3/INT6 easily interconvert each other through TS16 and TS17 (for the structures, see Figures S1 and S2 in the Supporting Information). For these species, the relative energy is very similar (see Figure 4), and for the isomerization processes, very small barriers were found. The two *syn-* and *anti-*conformers differ in energy by 6.4 kJ/mol, with the *syn-*form being the lower energy rotamer CH_2_CH_2_OH. This PES is the first where intermediate rotamers are distinguished. As already mentioned, we have explored the different reaction pathways leading to the two different CH_2_CHOH conformer products as well as to predict their BFs because spectroscopic techniques used to observe radicals and molecules in the ISM can readily distinguish both rotamers and conformers.^26^

The analysis of the PES reported in Figure 4 shows that the initial attack of the OH radical can proceed either via H abstraction, which yields CH_2_CH + H_2_O [channel (5)], or via the addition on the π-orbital of the double carbon–carbon bond of C_2_H_4_. Even though the CH_2_CH + H_2_O product channel is exothermic by 30.9 kJ/mol with respect to the reactants, the system needs to overcome an entrance barrier of 21.5 kJ/mol in order to form the products. In contrast, the addition route leads to the formation of a weakly bound vdW complex with C_2*v*_ point group symmetry, in which the reactants are perpendicular to each other, with the hydrogen of the OH radical pointing toward the C=C double bond (see Figure S2 in the Supporting Information). The vdW complex lies 8.3 kJ/mol below the reactant energy asymptote, while a “submerged” transition state (TS2) with a relative energy of −7.6 kJ/mol must be overcome in order to lead to the formation of the INT1 intermediate, where a new C–O bond is formed. Subsequently, the reaction can proceed in different directions. The intermediate INT1, located 109.5 kJ/mol below the reactant energy asymptote, might dissociate into *syn*-CH_2_CHOH and atomic hydrogen [channel (3)] by overcoming a barrier (TS6) of 140.5 kJ/mol (from INT1). This reaction channel is overall endoergic by 9.7 kJ/mol at our level of calculations. Alternatively, INT1 can isomerize into INT2 through a tight transition state (TS3) with a barrier height (from INT1) of 132.2 kJ/mol. The process involves a [1,3]-H transfer from the oxygen atom to the carbon atom. INT1 can also undergo a [1,2]-H shift by overcoming a barrier of 163.7 kJ/mol (TS4) to form INT3, which is 27.1 kJ/mol lower in energy than INT1 (see Figure 4). Finally, the hydrogen atom bound to the oxygen in INT1 can rotate by 180° through TS16 over a 3.9 kJ/mol barrier to form the other rotamer (INT5), which lies 107.6 kJ/mol below the reactants. Given the low TS, the two rotamers are expected to be in equilibrium. The fission of the C–H bond of INT5 leads to *anti*-CH_2_CHOH + H [channel (3)]. A barrier (TS10) of 121.8 kJ/mol (from INT5) must be overcome. Finally, INT5 can also isomerize to INT6 by overcoming a barrier of 160.5 kJ/mol (see below).

Once formed, INT2 can dissociate into CH_3_CHO + H [channel (2)] by overcoming a barrier (TS8) of 92.5 kJ/mol (with respect to INT2). The channel leading to CH_3_CHO + H products is exothermic by 34.4 kJ/mol and exhibits an exit barrier of 26.8 kJ/mol with respect to products. Alternatively, a competing channel leads to H_2_CO + CH_3_ [channel (1)] through the TS7 saddle point located −25.4 kJ/mol below the reactant energy asymptote. This is the most exothermic channel of the title reaction with a Δ*H*_0_^0^ = −53.5 kJ/mol. Other possible routes involve the isomerization of INT2. For instance, INT3 can be formed from INT2 by H migration from the carbon to the oxygen, a process that requires overcoming a barrier (from INT2) of 117.9 kJ/mol (TS5). Alternatively, INT2 can isomerize to INT4 by overcoming a very high barrier (TS13) lying 119.9 kJ/mol above the reactant energy asymptote. This isomerization process is highly unlikely.

From INT3, three products can be formed (see Figure 4). Formation of *syn*-CH_2_CHOH + H [channel (3)] through a barrierless process occurs via H-elimination from the methyl group in INT3. Another pathway follows the migration of H of the methyl group to the OH group, and the breaking of the C–O bond results in CH_2_CH + H_2_O [channel (5)]; however, the energetic barrier is so high (300.1 kJ/mol from INT3) that the process is unlikely to occur. Alternatively, CH_3_CHO + H [channel (2)] can also be formed from INT3 by an O–H bond fission overcoming a barrier (TS9) of 144.3 kJ/mol. At last, INT3 can isomerize into INT6 by rotating the O–H bond over a 12.2 kJ/mol barrier (TS17). The process can then be followed by the elimination of an H in the methyl group to form *anti*-CH_2_CHOH + H [channel (4)].

From INT4, the elimination of H from the methyl group results in *cyc*-CH_2_(O)CH_2_ + H. Since this is a highly endothermic product channel (Δ*H*_0_^0^ = 80.6 kJ/mol) formed by overcoming a very high barrier (TS14) (192.9 kJ/mol from INT4), the formation of *cyc*-CH_2_(O)CH_2_ + H does not occur unless the reaction takes place at very high energy (this is true for all this portion of the PES).

### 4.2 Branching Fractions

RRKM estimated of BFs have been done under the conditions of our CMB experiments (*E*_c_ = 50.4 kJ/mol) to assist the interpretation of the scattering data. We have also performed estimates under other conditions, considering *T* = 300, 400, 500, 1000, and 2000 K, which are of combustion and astrochemical interest (e.g., *T* of PDR regions is up to 1000 K, while *T* of circumstellar envelopes of late AGB stars is about 1000–2000 K).

Since the RRKM method can be applied only to the decomposition of bound intermediates, we could not account for the H-abstraction process, which is a direct process and does not proceed with the formation of intermediates, and the formation of H_2_O could not be included in the global branching fractions. The results of the calculated product BFs as a function of temperature are reported in Table 1, together with the BFs calculated at the experimental *E*_c_ of 50.4 kJ/mol and at the somewhat higher *E*_c_ value of 59.0 kJ/mol chosen to mimic the lowest value of excess energy (above the dissociation to the OH + C_2_H_4_ asymptote) of the CH_2_CH_2_OH intermediate produced in the photochemical experiment of Reisler and co-workers.^42^ In Table 1, also the derived product BFs obtained from the photochemical study of Reisler and co-workers^42^ and Butler and coworkers^44,45^ are reported, with indicated the excess energy of the chemically activated CH_2_CH_2_OH intermediate, photochemically produced in their experiment. In Table 1, the BFs calculated from the parameters of Table 6 (see also Figure 11) of^52^ (for *P* = 0 atm) for the same temperatures of our statistical calculations are also indicated within parentheses. The BFs for the two conformers of vinyl alcohol are distinct only in our calculations, while all the other literature values (from refs^42^,^44^,^45^ and^52^) represent the sum of the *anti*-CH_2_CHOH and *syn*-CH_2_CHOH BFs. It is interesting to note that the product BFs at *E*_c_ = 50.4 kJ/mol of our experiment correspond approximately to the values calculated for temperatures between 500 and 1000 K (being closer to the values at 500 K). We remind that we have been unable to determine experimentally BFs, while we have provided experimental evidence that vinyl alcohol is the major product formed by H-displacement.

**Table 1:** Calculated Product Branching Fractions (%) from the Addition Pathway for the OH + C_2_H_4_ Reaction at Different Temperatures and at the Experimental *E*_c_ = 50.4 kJ/mol and at *E*_c_ = 59.0 kJ/mol^a^

temperature (K)	***anti*-CH_2_CHOH + H (%)**	***syn*-CH_2_CHOH + H (%)**	**CH_3_CHO + H (%)**	**H_2_CO + CH_3_ (%)**
**300**	**0.14**	**0.22**	**1.70**	**97.94**
	(1.5)^52^		(5.5)^52^	(93.0)^52^
**400**	**2.27**	**2.83**	**2.60**	**92.29**
	(11.1)^52^		(10.6)^52^	(78.3)^52^
**500**	**9.18**	**10.04**	**3.36**	**77.42**
	(35.8)^52^		(10.8)^52^	(53.4)^52^
**1000**	**39.49**	**34.08**	**5.45**	**20.98**
	(87.3)^52^		(5.6)^52^	(7.1)^52^
**2000**	**44.64**	**36.58**	**6.60**	**12.18**
	(89.9)^52^		(7.9)^52^	(2.2)^52^
***E*_c_ = 50.4 kJ/mol**	**18.06**	**19.96**	**3.83**	**58.15**
***E*_c_ = 59.0 kJ/mol**	**25.63**	**25.99**	**3.86**	**44.42**
excess energy 60–95 kJ/mol^42^	dominant		very minor	−
excess energy 110–160 kJ/mol^44^	58		3	39
excess energy 110–160 kJ/mol^45^	29		0.001	71

a The BFs derived from the photochemical studies of refs (42),^44^ and^45^ are also indicated. The theoretical values in parentheses are calculated as a function of *T* from the parameters of Table 6 for *P* = 0 atm (collisionless limit) of.^52^ Since in the “collisionless limit”, the dominant product channel predicted in^52^ is the abstraction channel (5) (BF ∼ 90% from 300 to 2000 K), for a more direct comparison, we have normalized those BFs to 100% of the other (listed) four possible channels arising from the addition pathway, accounting for only about 10% of the total yield of the OH + C_2_H_4_ reaction.

In Table 2 we also report the comparison between the vinyl alcohol syn/anti ratio, as obtained from the present RRKM/ME results (Table 1), and from the equilibrium concentrations using the equilibrium constant estimated from known formulas as a function of temperature (see Supporting Information). As clearly visible, there is a significant difference that diminishes with the increase of the temperature. The results presented in Tables 1 and 2 will be discussed in Section 5.

**Table 2:** Ratios of *syn*-CH_2_CHOH/*anti*-CH_2_CHOH Products as Obtained from the Present Statistical RRKM/ME Calculations (Derived from the Data in Table 1) and from Equilibrium Concentrations of the Two Conformers (from the Equilibrium *syn*-CH_2_CHOH ⇔ *anti*-CH_2_CHOH, See Supporting Information), as a Function of Temperature

	*syn*-CH_2_CHOH/*anti*-CH_2_CHOH	
temperature (K)	RRKM/ME statistical ratio	equilibrium concentration ratio
300	1.57	5.17
400	1.25	3.11
500	1.09	2.28
1000	0.86	1.20
2000	0.82	0.87

## 5 Discussion

The reactive scattering signal at *m*/*z* = 46, corresponding to products with the gross formula C_2_H_4_^18^O from the neutral–neutral reaction of ^18^OH with C_2_H_4_ at *E*_c_ = 50.4 kJ/mol, can arise from the three H-displacement channels that are energetically open under our experimental conditions, namely channels (2), (3), and (4). Since these three reaction channels, leading to CH_3_CHO + H, *syn*-CH_2_CHOH + H, and *anti*-CH_2_CHOH + H, respectively, have similar reaction exothermicity and are characterized by a similar reaction mechanism, we could not disentangle their relative contributions (BFs) solely on the basis of the analysis of the experimental data, even though the cutoff of the *P*(*E*_T_′) distribution is more in line with a prevalent contribution from the CH_2_CHOH + H channels. The relative BFs were provided by RRKM statistical calculations on the PES that we have derived for this purpose (see Table 1). By combining the experimental observations and the theoretical predictions of product BFs, we can discuss the overall addition mechanism of the reaction.

Our RRKM results, reported in Table 1, show that at *E*_c_ = 50.4 kJ/mol, the dominant channel is the one associated with the decomposition of INT2 (see Figure 4) into H_2_CO + CH_3_ [channel (1)], with a value of branching fraction of 58.15%. This result can be explained by considering (see Figure 4) the lower energy barrier (represented by TS3) related to the isomerization of INT1 to INT2, with respect to the H-elimination processes, whose transition states (TS6 and TS10) are, with respect to TS3, respectively 8.3 and 9.6 kJ/mol higher in energy. The second and third most important channels are those associated with the fission of a C–H bond from INT1 and INT5, leading to the formation of *syn-*CH_2_CHOH + H and *anti-*CH_2_CHOH + H [channels (3) and (4)] with BFs of 19.96 and 18.06%, respectively. The sum of these two channels leads to a global value of BF for the formation of vinyl alcohol of 38.02%. Finally, the last channel [channel (2)] related to the formation of acetaldehyde + H shows a value of BF of only 3.83%. This can be explained by the fact that channel (2) originates from the same INT2 intermediate and that the C–C sigma bond is weaker than the C–H bond.

Significant variations of the product BFs can be appreciated with increasing temperature. At 300 K, the dominant addition channel is that leading to H_2_CO + CH_3_ (BF = 97.94%), followed by CH_3_CHO + H, which is minor (BF = 1.70%); at this temperature, the BFs for *syn*- and *anti*-vinyl alcohol are even smaller (global BF = 0.36%). For *T* < 300 K (values not reported in Table 1), the BF of H_2_CO + CH_3_ is even larger than at 300 K, and comparatively, the ratio (acetaldehyde)/(vinyl alcohol) increases further, but the overall H-displacement channels become negligible with respect to H_2_CO + CH_3_. This is in agreement with the predictions of^52^ (see BF values in parenthesis). With the increase of the temperature, the trend is reversed. In fact, for temperatures higher than 400 K, the formation of vinyl alcohol is favored, with a global *syn-*/*anti-* BF of 5.10% with respect to acetaldehyde (BF = 2.60%) at 400 K, and 19.22% vs 3.36% at 500 K (see Table 1). Finally, for temperatures ≥1000 K, the main product channels appear to be the formation of *syn*- and *anti*-vinyl alcohol [channels (3) and (4)], with BFs of 34.08 and 39.49%, respectively, leading to a global value of BF for vinyl alcohol of 73.57% at 1000 K. The second most abundant channel at 1000 K is the formation of formaldehyde + CH_3_ [channel (2)], with a BF of 20.98%, while the formation of acetaldehyde + H [channel (2)] accounts for only 5.45% of the total addition reactive flux. Therefore, an increase of the energy available to the system allows it to overcome higher barriers and leads to an appreciable amount of less exothermic products which, in turn, are disfavored at low temperatures. Finally, by increasing the temperature to 2000 K, a further increase of the values of BFs for the H-elimination processes (BF = 44.64, 36.58, and 6.60% for *anti*-CH_2_CHOH, *syn*-CH_2_CHOH, and CH_3_CHO + H, respectively) can be noticed, while the fraction of formaldehyde + CH_3_ decreases (BF = 12.18%).

As previously illustrated, the first step of the reaction is represented by the formation of a vdW complex followed by the formation, via a submerged barrier, of a new C–O bond in a more stable, covalently bound intermediate INT1/INT5 (Figure 4). Our theoretical results indicate that the most probable fate of INT1/INT5 is by back-dissociation to reactants, which represents at least 85% of the initial flux, a frustrated reactive flux that does not evolve to bimolecular products [channels (1–4)]. The reason of the high fraction of back-dissociation of INT1 (and its isomer INT5) is due to the absence of a barrier higher than the reactant asymptote in the back direction between INT1 and the vdW well (in fact, TS2 is a submerged barrier), while the evolution of INT1/INT5 to products can only occur via barriers (TS3, TS6, TS10) located above the reactant asymptote (see Figure 4). The reactive flux is then distributed among the possible products [channels (1–4)]. As can be seen from the PES (Figure 4), once formed, the two isomers INT1 and INT5 can directly dissociate to form vinyl alcohol [channels (3) and (4)] or, competitively, isomerization can take place, leading to the formation of INT2. A subsequent C–C fission leads to the formation of formaldehyde together with the CH_3_ radical [channel (1)] or, alternatively, a new H-elimination process leads to the formation of acetaldehyde + H [channel (2)].

It should be noted that our calculated BF values (Table 1) are in agreement with the fact that the vinyl alcohol BF is growing with the rise of the temperature, as predicted in the seminal theoretical study of.^52^ The trends of our BFs as a function of *T* agree with those predicted in,^52^ also for the CH_3_CHO + H and H_2_CO + CH_3_ channels. Furthermore, our results (see Table 1, values at *E*_c_ = 59.0 kJ/mol) are in agreement with the conclusions of Reisler and co-workers^42^ that, for a comparable excess energy of the initial reaction CH_2_CH_2_OH intermediate (2-hydroxyethyl radical), the BF of the vinyl alcohol + H channel is much larger than that of acetaldehyde + H. Good qualitative agreement is also noted with the product BFs derived by Butler and co-workers^44,45^ at a somewhat higher excess energy; in particular, our RRKM predictions that the global BF of *syn*-/*anti*-vinyl alcohol is comparable to the BF of H_2_CO + CH_3_ and much larger than that of acetaldehyde at *E*_c_ = 50.4 kJ/mol are in line with those from the most recent photochemical study with synchrotron radiation.^45^

The BF predicted by Senosiain et al.^52^ for the H_2_CO + CH_3_ channel decreases with increasing *T* somewhat faster than the BF predicted in the present study. Conversely, the BF for vinyl alcohol formation increases somewhat faster with *T* than our global (anti + syn) BF. These differences can be mainly attributed to small differences in barrier eights that, however, fall nearly within the accuracy of the theoretical methods used to calculate them. Overall, our statistical predictions are in agreement with those of Senosiain et al.,^52^ both qualitatively in their trend with the increase of *T* from 300 to 2000 K and nearly quantitatively in their values.

Our results indicate that the slightly more exothermic syn-conformer channel has a slightly larger BF at all temperatures (about 10% larger at the experimental *E*_c_ of 50.4 kJ/mol). The ratio syn/anti represents useful information in astrochemical applications since the spectroscopic techniques employed to detect vinyl alcohol in the ISM are conformer-specific, as discussed in the Introduction and next. Since the kinetic *syn*/*anti*-vinyl alcohol product ratios and their dependence on *T* are substantially different from the ratios derived from the equilibrium concentrations (as can be seen from Table 2, the equilibrium ratio at 300 K is 3.3 times larger than the kinetic ratio, while, decreasing faster with increasing *T*, it becomes nearly equal to—only a factor 1.06 larger than—the kinetic ratio at 2000 K), the measurements of this ratio in various environments could be used as an indicator that their chemistry is determined either by thermodynamics or kinetics.

## 6 Implications for Astrochemistry

More than 270 interstellar/circumstellar molecules have been detected by astronomical observations to date.^5^ The OH radical is one of the most abundant radicals in space, being generated mostly by its parent species H_2_O. It has been detected in many extraterrestrial environments, like planetary atmospheres,^7,8^ comets,^9^ young stellar objects, photon-dominated regions, and protoplanetary disks.^10−12^ Ethylene is the simplest alkene and is expected to be present in many interstellar environments. Because of the lack of a permanent dipole moment, however, there are very few detections in emission, and these are limited to the circumstellar envelope of the AGB star IRC+10216.^13,14^ However, ethylene is considered in all chemical networks of reactions.^105,106^ Since acetaldehyde and vinyl alcohol can be formed in the OH + C_2_H_4_ reaction, a verification of whether the title reaction is a viable formation route of acetaldehyde and/or vinyl alcohol in warm or hot regions of the ISM is of considerable interest. There is a third isomer with the same C_2_H_4_O gross formula, that is, oxyrane which has also been widely detected in the ISM.^5^ Among the three C_2_H_4_O isomers, vinyl alcohol has been the last one to be detected. Positive detection toward SgrB2 dates to 2001.^4^ More recent attempts by Melosso et al.,^25^ who searched for vinyl alcohol in the ASAI spectral line survey, were unsuccessful. A clear detection of *syn*-vinyl alcohol has been recently achieved by Agúndez et al.,^26^ who also detected several other O-bearing complex organic molecules toward the starless core TMC-1. Concerning interstellar acetaldehyde, a recent critical overview of the possible formation routes points to the reactions O + C_2_H_5_ and CH_3_CHOH + O → CH_3_CHO + OH as the most relevant pathways in the gas phase.^24^ Vinyl alcohol (C_2_H_3_OH) has never been specifically considered in astrochemical models. It is reasonable that some of the reactions that are considered to produce CH_3_CHO could also form C_2_H_3_OH. For instance, there is experimental evidence that the CCO backbone is preserved with a branching fraction of 23%^107^ in the dissociative electron recombination of CH_3_CHOH^+^ that could bring to both CH_3_CHO and C_2_H_3_OH. Experimental evidence of C_2_H_3_OH was given in proton irradiation of H_2_O/C_2_H_2_ ices,^108^ electron irradiation of CO/CH_4_ and H_2_O/CH_4_ ices^109−111^ while non-energetic processing of C_2_H_2_ ices where H atoms and OH radicals were deposited on the surface also produced C_2_H_3_OH.^112^ Finally, a theoretical characterization of the C_2_H reaction directly with the water molecules of the ice substrate indicated a very efficient route toward vinyl alcohol formation and, by successive hydrogenation, to ethanol.^113^ We recall that ethanol detection on interstellar ice has been the first achievement of the James Webb Space Telescope as far as new detection of interstellar icy molecules is concerned.^114^ Therefore, there are processes that could form C_2_H_3_OH on the surface of interstellar grains but its detection for the first time in a cold cloud at very low temperature^26^ points to a gas-phase formation route because it is unclear how vinyl alcohol formed on ice could then be released in the gas phase at those very low *T* (like all the other alcohols, it is expected to have a large binding energy with the ice surface).

Taking into account the high exit potential barriers and the enthalpies of the overall reaction, the present study suggests that the OH + C_2_H_4_ reaction is a possible formation route of *syn*-/*anti*-vinyl alcohol only in the warm regions of the ISM (circumstellar envelopes, PDR) because these reaction channels are slightly endothermic and the minimum energy path leading to their formation is characterized by transition states which are somewhat above the reactant’s energy. However, in the cold regions of the ISM, as TMC-1, stabilization of the initial CH_2_CH_2_OH intermediate by radiative association can be a significant outcome of the title reaction. While radiative association has been considered for many ion-molecule processes,^115^ it has been much less explored for neutral–neutral reactions. Yet, there is at least one case, the reaction of atomic oxygen with benzene, where the formation of phenol by radiative association has been verified under single collision conditions.^116−118^ Also, radiative association for the reaction H + C_6_H_5_ has been considered^119^ in the chemistry of the upper atmosphere of Titan, while, more recently, it has been demonstrated that the radiative association of CH_3_ + CH_3_O can actually lead to dimethyl ether in a very fast process,^120^ as suggested by Balucani et al.^121^ The requisites for a radiative association process to be possible are that no easy exothermic channels with bimolecular products are available and that there are a large number of internal degrees of freedom to increase the lifetime of the intermediate. In this respect, we note that the number of atoms of the 2-hydroxyethyl radical is smaller by only one unit with respect to that of dimethyl ether and that, once formed, CH_2_CH_2_OH is trapped in a potential well located at −109.5 kJ/mol (see Figure 4). To evolve further, it needs to overcome a barrier (the lowest one is at 132.2 kJ/mol from the bottom of the INT1 well), and, indeed, the 2-hydroxyethyl radical is seen to dissociate back to OH + C_2_H_4_ even when it is formed with a very large amount of internal energy (see the experiments by Reisler and co-workers^42^ and Butler and co-workers^44,45^). Furthermore, collisional stabilization has been observed in kinetic experiments, also in those performed at very low pressure. This can be taken as a general indication that stabilization can be an easy process. In this hypothesis, the title reaction could be a really significant source of 2-hydroxyethyl radical, a species which is claimed to be a very important intermediate toward the formation of glycolaldehyde.^99^ In particular, this reaction could help in those conditions where ethanol is not an abundant species because it is still trapped in interstellar ice. Furthermore, radiative association leading to the 2-hydroxyethyl radical could be the missing step in vinyl alcohol formation in cold objects: once stabilized, the CH_2_CH_2_OH radical can be converted to vinyl alcohol via H-abstraction reactions by a plethora of radicals, including OH, CN, or F, Cl, and H atoms. The reactions of H atoms with the isomers of 2-hydroxyethyl (ethoxy and 1-hydroxyethyl radicals)^122^ have been explored theoretically, while for the CH_2_CH_2_OH radical reaction, an experimental value of 8.3 × 10^–11^ cm^3^ s^–1^ has been inferred by Bartels et al.^41^ They were unable to determine which product of the gross formula C_2_H_4_O was formed, but the abstraction of one H atom bound to the same carbon atom holding the −OH group is favored. In conclusion, radiative association to the CH_2_CH_2_OH radical could explain the formation of vinyl alcohol in very cold object as well as to contribute to the formation of glycolaldehyde. To address this point, we have estimated the rate for the radiative association for the CH_2_CH_2_OH radical as a function of the temperature by modifying our home-made code, taking into account the possible formation mechanism of the initial INT1 intermediate, the possibility of its back-dissociation to the reactants and its radiative stabilization, that we have calculated in accordance with the model proposed by Herbst,^120,123^ using the Einstein coefficients for spontaneous emission, calculated considering the intensity of the vibrations of the INT1 intermediate. We found a value at the temperature of 10 K for the radiative association rate (*k*_rad-ass_) of ∼1 × 10^–12^ cm^3^ mole c^–1^ s^–1^. This finding tends to support the possibility that radiative association to the CH_2_CH_2_OH radical from the OH + C_2_H_4_ reaction could explain the formation of vinyl alcohol in very cold objects as well as to contribute to the formation of glycolaldehyde.

## 7 Conclusions

The addition of OH(^2^Π) to C_2_H_4_ was investigated by exploiting the CMB technique with mass spectrometric detection and TOF analysis at the collision energy of 50.4 kJ/mol. Products attributable to the three energetically open H-displacement channels were observed. The underlying reaction mechanisms were unraveled through the combination of the experimental results with ab initio electronic structure calculations of the PES and statistical RRKM computations of product branching fractions. The title reaction was found to proceed indirectly (that is, via a long-lived complex mechanism) and is initiated by the barrierless addition of OH on the π-orbital of the double carbon–carbon bond in the C_2_H_4_ molecule, forming a long-lived CH_2_CH_2_OH intermediate, which proceeds through hydrogen elimination to *syn*-CH_2_CHOH or undergoes further isomerization processes and ultimately fragment to *anti*-CH_2_CHOH and CH_3_CHO by hydrogen loss via a tight exit transition state. RRKM calculations of product distributions have determined that the acetaldehyde-forming channel is prioritized over the vinyl alcohol-forming channels when the temperature is below 300 K, while the dominant hydrogen loss channels correspond to *syn*-CH_2_CHOH + H and *anti*-CH_2_CHOH + H under our experimental conditions (and in general for *T* > 500 K). Since the formation of *syn*-CH_2_CHOH and *anti*-CH_2_CHOH is endoergic by 5.2(9.7) and 9.6(14.2) kJ/mol experimentally(theoretically), respectively, taking into account the high exit potential barriers and the overall reaction enthalpies, the OH + C_2_H_4_ reaction is likely a possible formation route to *syn*-/*anti*-vinyl alcohol in combustion processes and of both *syn*-/*anti*-vinyl alcohol in the warm regions of the ISM (circumstellar envelopes, PDR). It is suggested that the radiative association of OH and C_2_H_4_ to the CH_2_CH_2_OH radical could explain the formation of *syn*-/*anti*-vinyl alcohol also in very cold objects (such as TMC-1, where recently *syn*-vinyl alcohol has been observed^26^).

Overall, the present experimental/theoretical work establishes that *syn*-/*anti*-vinyl alcohol is a substantial product of the OH + C_2_H_4_ reaction also under single-collision conditions and elucidates the dynamics of formation of the two conformers of the vinyl alcohol product. Since the kinetic *syn*-/*anti*-vinyl alcohol ratio is quite different (3.3 times smaller at 300 K and 1.06 times smaller at 2000 K) with respect to that associated with thermodynamic equilibrium, its value can be taken as an indication that the chemistry of a certain medium is kinetically controlled or not.

The present theoretical predictions of product BFs as a function of temperature for the title reaction via the addition mechanism are in line the previous similar predictions of the detailed theoretical study of Senosiain et al.^52^ Finally, the formation of vinyl alcohol via the OH + C_2_H_4_ reaction should be included not only in combustion flames,^52,68^ but also in astrochemical models where vinyl alcohol has never been specifically considered.

## Supporting Information Available

Enthalpy changes and barrier heights computed for selected dissociation and isomerization processes of the OH + C_2_H_4_ reaction; calculated energy barriers of the OH + C_2_H_4_ reaction; geometries of the reactants and products, of the van der Waals adduct, of the intermediates, and of the transition states along the PES; Cartesian coordinates and vibrational frequencies for all stationary points of the PES; and method of calculation of the equilibrium concentration ratios between the *syn*-/*anti*-CH_2_CHOH rotamers (PDF)
