# Supplementary material for: OH(2Π) + C2H4 Reaction: A Combined Crossed Molecular Beam and Theoretical Study
Source: J Phys Chem A. 2023 May 19;127(21):4609–23. doi: 10.1021/acs.jpca.2c08662 (PMC10240494; doi:10.1021/acs.jpca.2c08662)
Supplement: Supplementary file 1 — jp2c08662_si_001.pdf [file jp2c08662_si_001.pdf]

Supporting Information (SI) for the paper

The $OH(^2\Pi)$ + C_2H_4 Reaction: A Combined Crossed Molecular Beam and Theoretical Study

Pengxiao Liang[a], Emília Valença Ferreira de Aragão[a,b], Lisa Giani[a,c], Luca Mancini[a], Giacomo Pannacci[a], Demian Marchione[a], Gianmarco Vanuzzo[a], Noelia Faginas-Lago[a,b], Marzio Rosi[d], Dimitrios Skouteris[b], Piergiorgio Casavecchia,[a,*] Nadia Balucani[a,*]

[a]*Dipartimento di Chimica, Biologia e Biotecnologie, Università degli Studi diPerugia, Perugia, 06123, Italy*

[b]*Master-Tec srl, Via Sicilia, 41, Perugia, 06128, Italy*

[c]*Université Grenoble Alpes, 621 Av. Centrale, 38400 Saint-Martin-d'Hères, France*

[d]*Dipartimento di Ingegneria Civile ed Ambientale, Università degli Studi diPerugia, Perugia, 06125, Italy*

Table S1. Enthalpy changes and barrier heights (kJ/mol, 0 K) computed at the CCSD(T)/aug-cc-pVTZ level of theory for selected dissociation and isomerization processes for the OH($^2\Pi$) + C_2H_4 reaction.

	ΔH^0_0	Barrier height
CH_2CH_2 + OH → vdW	−8.3	-
CH_2CH_2 + OH → CH_2CH + H_2O	−30.9	21.5
vdW → INT1	−101.2	0.8
INT1 → INT2	9.4	132.2
INT1 → INT3	−27.1	163.7
INT1 → INT5	1.9	3.9
INT1 → *syn*-CH_2CHOH + H	119.5	140.5
INT2 → INT3	−36.5	117.9
INT2 → INT4	17.3	220.0
INT2 → CH_3CHO + H	65.7	92.5
INT2 → H_2CO + CH_3	46.6	74.7
INT3 → CH_3CHO + H	102.2	144.3
INT3 → *syn*-CH_2CHOH + H	146.3	-
INT3 → CH_2CH + H_2O	105.7	300.1
INT3 → INT6	−1.3	12.2
INT4 → H_2CO + CH_3	29.3	109.7
INT4 → cyc-$CH_2(O)CH_2$ + H	163.4	250.3
INT5 → INT6	−30.3	160.5
INT5 → *anti*-CH_2CHOH + H	121.8	139.9
INT6 → *anti*-CH_2CHOH + H	152.1	-

Table S2: Calculated energy barriers (in kj/mol) in theoretical studies of the OH($^2\Pi$) + C_2H_4 reaction.

	This work[a]	Ref. (1)[b]	Ref. (2)[c]	Ref. (3)[d]	Ref. (4)[e]	Ref. (5)[f]
CH_2CH_2+OH → CH_2CH+H_2O	21.5	20.5	24.7		27.6	
CH_2CH_2 + OH → INT1			−3.3		−9.2	−2.1
vdW → INT1	0.8	8.8	4.6		0.4	
INT1 → INT2	132.2	133.5	142.3		130.5	
INT1 → INT3	163.7	160.7	157.7		165.7	
INT1 → INT5	3.9					
INT1 → s-CH_2CHOH + H	140.5	138.5	133.5		131.8	
INT2 → INT3	117.9	114.2		112.5	125.5	
INT2 → INT4	220.0					
INT2 → CH_3CHO + H	92.5	89.1			86.2	
INT2 → H_2CO + CH_3	74.7	72.8	57.7		74.1	
INT3 → CH_3CHO + H	144.3	146.4		136.4		
INT3 → s-CH_2CHOH + H		151.9				
INT3 → CH_2CH + H_2O	300.1					
INT3 → INT6	12.2					
INT4 → H_2CO + CH_3	109.7					
INT4 → cyc-$CH_2(O)CH_2$ + H	250.3					
INT5 → INT6	160.5					
INT5 → a-CH_2CHOH + H	139.9					

[a]CCSD(T)//B3LYP/aug-cc-pVTZ.
[b]RQCISD(T)/cc-pV∞Z//UQCISD/6-311++G(d,p).
[c]PMP2/aug-cc-pVQZ//MP2/cc-pVTZ.
[d]QCISD(T)/aug-cc-pVTZ // MPW1K/6-31+G(d,p).
[e]QCISD(T)/6-311G(2df,p)//B3LYP/6-311G(d,p).
[f]PMP2/aug-cc-pVTZ//MP2/6-311G**.

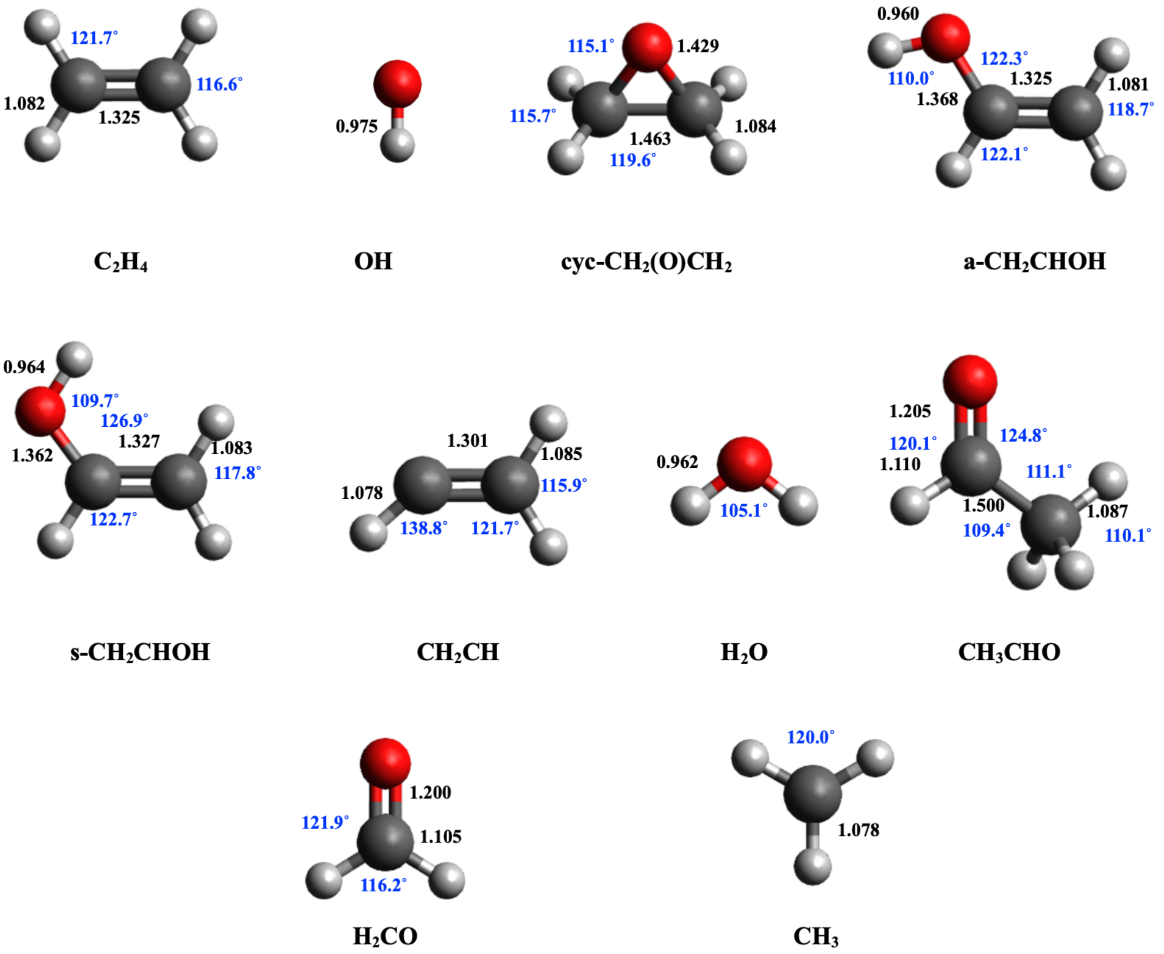

Figure S1. Geometries of the reactants and of the products of the $OH(^2\Pi)$ + C_2H_4 reaction optimized at the B3LYP/aug-cc-pVTZ level of theory. Interatomic distances (in Å) are displayed in black and angles (in degrees) in blue. Oxygen, carbon and hydrogen atoms are represented in red, grey, and white, respectively.

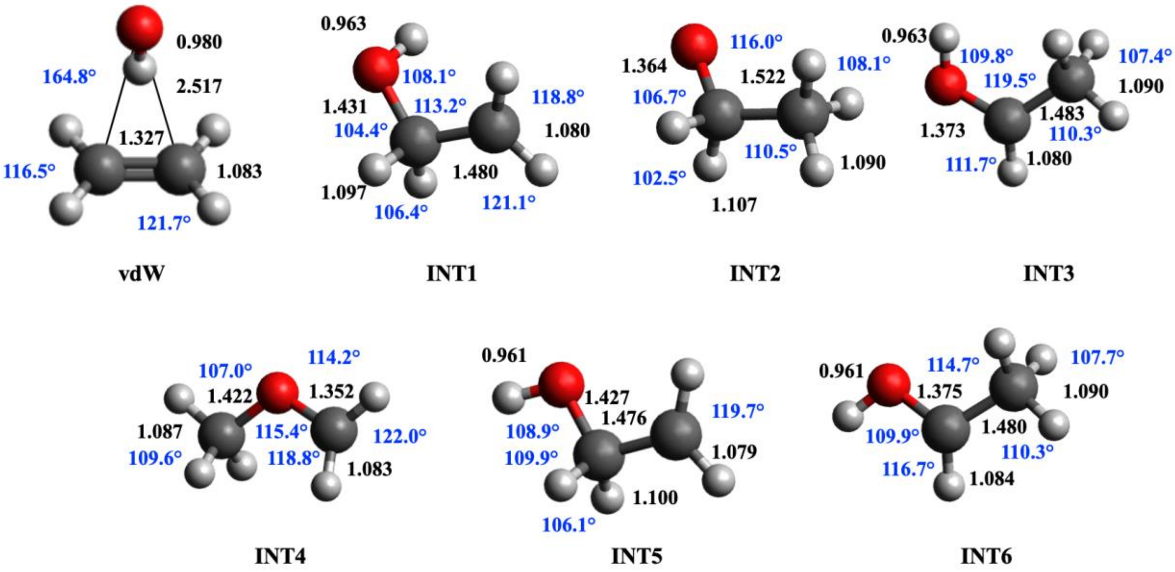

Figure S2. Geometries of the vdW adduct and intermediates identified along the PES for the OH($^2\Pi$) + C_2H_4 reaction at B3LYP/aug-cc-pVTZ level of theory. Interatomic distances (in Å) are displayed in black and angles (in degrees) in blue. Oxygen, carbon and hydrogen atoms are represented in red, grey, and white, respectively.

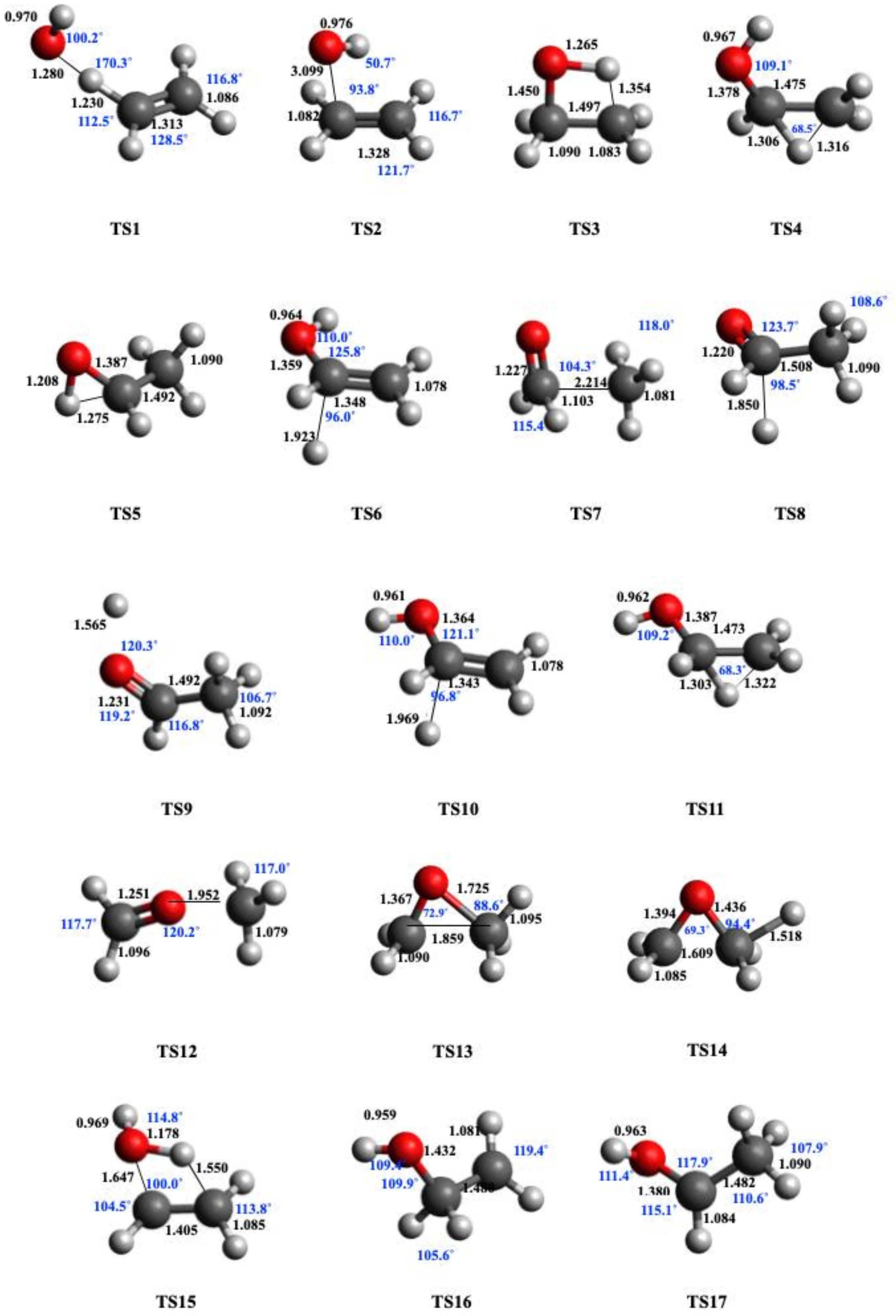

Figure S3. Transition state geometries identified at the B3LYP/aug-cc-pVTZ level of theory in the PES of the OH($^2\Pi$) + C_2H_4 reaction. Interatomic distances (in Å) are displayed in black and angles (in degrees) in blue. Oxygen, Carbon and Hydrogen atoms are represented in red, grey, and white, respectively.

Table S3. Cartesian coordinates and vibrational frequencies for all the stationary points identified at the B3LYP/aug-cc-pVTZ level of theory in the PES of the OH($^2\Pi$) + C_2H_4 reaction.

**Stationary Point**	**Cartesian Coordinates**				**Vibrational Frequencies (cm^{-1})**
**C_2H_4**	C	0.00000	0.00000	0.66241	834.3297, 977.0322, 983.9029, 1059.4457, 1245.7248, 1380.5384 1478.5981, 1688.9350, 3128.4046 3142.1096, 3197.7044, 3225.9716
	C	0.00000	0.00000	-0.66241	
	H	0.00000	0.92097	1.23071	
	H	0.00000	-0.92097	1.23071	
	H	0.00000	0.92097	-1.23071	
	H	0.00000	-0.92097	-1.23071	
**OH**	O	0.00000	0.00000	0.10837	3694.9818
	H	0.00000	0.00000	-0.86694	
**vdW**	C	-1.18562	0.66392	0.00092	74.1102, 92.4432, 104.6856 280.1450, 363.2749, 835.8523 997.2174, 998.7286, 1069.3378 1246.3139, 1380.4813, 1481.9085 1683.6250, 3127.9019, 3139.9679 3197.9803, 3225.7884, 3600.6875
	C	-1.18752	-0.66330	0.00092	
	H	-1.20126	1.23323	-0.91967	
	H	-1.17920	1.23330	0.92160	
	H	-1.20464	-1.23255	-0.91969	
	H	-1.18258	-1.23265	0.92161	
	O	2.22069	-0.00047	-0.00056	
	H	1.24097	-0.00132	-0.01043	
**INT1**	C	1.23496	-0.26965	0.00706	174.5363, 320.7903, 424.2156 544.9585, 824.9134, 946.7476 1076.9431, 1119.5211, 1179.4344 1354.2728, 1394.6940, 1455.0576 1483.8091, 2971.4678, 2990.8472 3141.1059, 3246.1512, 3799.9716
	C	-0.00458	0.53833	-0.03220	
	H	1.29503	-1.18534	-0.56465	
	H	2.12478	0.09745	0.49755	
	H	-0.02286	1.25643	0.79642	
	H	-0.06360	1.12834	-0.95551	
	O	-1.19518	-0.25554	-0.04081	
	H	-1.15417	-0.86468	0.70351	
**INT2**	C	0.17903	0.47487	-0.00038	72.0456, 251.6394, 435.5256 860.6978, 885.2488, 1065.6999 1097.0241, 1237.9452, 1328.3129 1389.8138, 1408.7504, 1485.5125 1494.8466, 2881.0465, 2885.6926 3032.1423, 3095.5476, 3105.8598
	O	1.25580	-0.36292	-0.00048	
	C	-1.18812	-0.19467	-0.00001	
	H	0.30002	1.16158	-0.85954	
	H	0.30278	1.15249	0.86616	
	H	-1.30699	-0.81947	-0.88515	
	H	-1.98358	0.55280	0.00335	
	H	-1.30413	-0.82526	0.88137	
**INT3**	C	1.20839	-0.18004	0.01876	191.2399, 328.5343, 409.5352 555.4606, 914.0490, 1016.1486 1059.4585, 1196.6228, 1301.4764 1400.4965, 1432.6106, 1466.7425 1483.6974, 2928.6530, 3002.8501 3093.9130, 3193.9031, 3798.3076
	C	-0.09050	0.52515	-0.10798	
	H	1.28087	-1.02272	-0.67786	
	H	2.03108	0.49893	-0.20387	
	H	-0.20807	1.56150	0.17256	
	O	-1.25973	-0.17710	0.04535	
	H	-1.10889	-1.10853	-0.14808	
	H	1.37552	-0.58308	1.02973	

Stationary Point	Cartesian Coordinates	Vibrational Frequencies (cm^{-1})
**INT4**	C 1.20394 0.22458 0.05946<br>H 1.13688 1.27559 -0.19220<br>H 2.12653 -0.32594 -0.02695<br>O 0.09232 -0.53957 -0.03326<br>C -1.14063 0.16783 0.01124<br>H -1.93176 -0.56499 -0.12244<br>H -1.18879 0.90898 -0.79143<br>H -1.26134 0.66840 0.97487	154.7898, 289.6219, 428.7123<br>513.1487, 954.0562, 1133.6399<br>1168.6932, 1247.4253, 1283.1288<br>1457.0062, 1489.2083, 1493.8316<br>1503.2329, 3004.5216, 3059.1148<br>3116.8381, 3130.0874, 3262.9309
**INT5**	C -1.25239 -0.25429 -0.01317<br>C 0.00394 0.51906 0.02791<br>H -1.24249 -1.30906 0.21477<br>H -2.19336 0.24865 -0.17693<br>H 0.03245 1.24395 -0.79879<br>H 0.06009 1.11153 0.95774<br>O 1.11357 -0.37583 -0.04569<br>H 1.92540 0.12297 0.08025	98.7091, 269.4503, 416.6000<br>464.0259, 866.3435, 953.4726<br>1058.0171, 1103.2444, 1217.8770<br>1264.2539, 1418.5747, 1458.2746<br>1487.5322, 2888.3918, 2942.3207<br>3156.9598, 3265.0840, 3827.7042
**INT6**	C 1.22820 -0.16303 0.01060<br>C -0.08877 0.50330 -0.09248<br>H 1.31083 -0.98116 -0.70923<br>H 2.02836 0.54963 -0.18706<br>H -0.23554 1.52831 0.22822<br>O -1.16979 -0.33919 0.01935<br>H -1.98478 0.17048 -0.00216<br>H 1.40286 -0.59535 1.00672	177.2322, 355.8982, 407.4438<br>537.7010, 926.8992, 1019.0230<br>1057.3616, 1203.6374, 1269.3984<br>1395.2961, 1444.3336, 1463.2493<br>1487.6222, 2944.8785, 3035.5043<br>3097.0553, 3139.0862, 3826.8689
**TS1**	C -1.50095 -0.37939 0.00642<br>C -0.58378 0.56028 -0.01341<br>H -2.55545 -0.13220 0.08826<br>H -1.25034 -1.43000 -0.06906<br>H -0.71530 1.63176 0.06686<br>H 0.59563 0.23998 -0.15611<br>O 1.81472 -0.14436 -0.09424<br>H 1.91606 -0.24007 0.86587	1129.8908*i*, 135.0360, 160.5294<br>307.1869, 604.6395, 792.3890<br>834.1774, 890.9585, 968.2445<br>1129.7799, 1221.9192, 1271.4367<br>1409.9150, 1666.5554, 3105.0143<br>3176.4122, 3194.1151, 3746.0060
**TS2**	C 0.91463 -0.70258 0.02516<br>C 1.33288 0.55757 0.01798<br>H 0.96707 -1.32478 -0.85882<br>H 0.51044 -1.16262 0.91756<br>H 1.74862 1.01383 -0.87152<br>H 1.28639 1.17656 0.90481<br>O -2.08462 0.07753 0.00378<br>H -1.32062 0.54681 -0.38114	147.3611*i*, 53.7271, 93.6902<br>173.2401, 186.6278, 834.8901<br>991.3620, 998.5623, 1063.6490<br>1246.4288, 1378.1971, 1479.9710<br>1678.7202, 3128.1231, 3140.6015<br>3199.1057, 3226.8304, 3676.6099
**TS3**	C -1.04954 -0.20656 0.00002<br>C 0.14522 0.69470 0.00004<br>H -1.59706 -0.33623 0.92496<br>H -1.59757 -0.33555 -0.92475<br>H 0.28174 1.29994 -0.89581<br>H 0.28178 1.29994 0.89584<br>O 1.01474 -0.46571 -0.00002<br>H -0.06094 -1.13123 -0.00041	1962.1562*i*, 383.2803, 713.0940<br>822.3644, 918.2933, 975.2265<br>1076.6603, 1081.4287, 1151.5142<br>1218.1967, 1308.8987, 1431.1504<br>1527.9640, 1921.0732, 3045.5224<br>3091.1744, 3115.2140, 3222.3183

Stationary Point	Cartesian Coordinates	Vibrational Frequencies (cm^{-1})
**TS4**	C -1.23029 -0.23754 -0.03444<br>C 0.04670 0.49860 0.02571<br>H -1.23584 -1.27965 0.24884<br>H -2.07355 0.17107 -0.57128<br>H 0.07875 1.50747 -0.36512<br>H -0.80770 0.43898 1.01226<br>O 1.25109 -0.17135 0.01664<br>H 1.13117 -1.03343 -0.40547	1872.5769*i*, 261.5566, 410.6081<br>439.9440, 687.3202, 768.4142<br>922.8158, 1074.3276, 1200.8958<br>1279.9986, 1312.8735, 1395.5963<br>1433.7060, 2137.0153, 3137.0378
**TS5**	C -0.12444 0.47549 0.02871<br>O -1.25831 -0.31512 -0.08956<br>C 1.21924 -0.17334 0.00234<br>H -0.97972 0.24219 0.94564<br>H -0.21684 1.49242 -0.35571<br>H 1.23820 -1.06863 0.62275<br>H 1.99237 0.51714 0.34048<br>H 1.46362 -0.47506 -1.02305	1960.5111*i*, 188.8534, 429.8814<br>601.7789, 868.9826, 901.4961<br>1062.7986, 1114.3163, 1172.6914<br>1358.7766, 1405.2744, 1471.3111<br>1479.6292, 2370.2277, 2994.7826<br>3056.5097, 3069.5743, 3110.7838
**TS6**	C -1.19577 -0.27330 0.01767<br>C -0.02610 0.37966 0.16668<br>H -1.24295 -1.28595 -0.36082<br>H -2.12402 0.24416 0.20049<br>H -0.16465 1.41289 -1.44937<br>H 0.03092 1.35430 0.62930<br>O 1.21123 -0.16452 0.02835<br>H 1.14207 -1.04737 -0.35250	719.4878*i*, 385.5661, 436.5182<br>439.3051, 496.8537, 674.6653<br>816.3988, 956.7617, 1020.1780<br>1112.6046, 1313.5110, 1337.1358<br>1447.7294, 1607.7606, 3149.0411<br>3196.7323, 3249.3681, 3789.6625
**TS7**	C 0.57456 0.54766 -0.00001<br>O 1.24312 -0.48151 0.00000<br>C -1.52197 -0.16433 0.00000<br>H 0.37961 1.10356 -0.93240<br>H 0.37962 1.10357 0.93238<br>H -1.49759 -0.72248 -0.92313<br>H -2.02542 0.79214 0.00136<br>H -1.49673 -0.72463 0.92181	322.9507*i*, 121.5489, 267.7001<br>498.6449, 537.9262, 589.5536<br>891.4469, 1099.7230, 1241.5049<br>1413.8102, 1427.1974, 1481.2999<br>1627.9954, 2897.0189, 2947.2036<br>3096.6640, 3258.0905, 3270.3458
**TS8**	C 0.22342 0.30009 0.22151<br>O 1.21747 -0.34140 -0.07673<br>C -1.18552 -0.16586 -0.04670<br>H 0.32366 1.62209 -1.06907<br>H 0.31626 1.17628 0.89523<br>H -1.20927 -0.86029 -0.88361<br>H -1.85719 0.67320 -0.22617<br>H -1.54060 -0.68544 0.84866	782.7798*i*, 170.7535, 379.8551<br>439.7085, 503.1324, 807.9645<br>900.0652, 1100.3950, 1127.8533<br>1378.2740, 1400.1909, 1462.2913<br>1474.2560, 1686.2074, 2864.2164<br>3022.4877, 3087.5727, 3133.2180
**TS9**	C -1.21818 0.20066 -0.01915<br>C 0.11500 -0.46553 0.05810<br>H -1.15333 1.14734 -0.55061<br>H -1.94873 -0.45419 -0.49800<br>H 0.12848 -1.51051 0.40778<br>O 1.18122 0.10302 -0.17470<br>H 1.72481 1.19450 0.80679<br>H -1.58193 0.38793 0.99794	1128.4677*i*, 98.1961, 155.2448<br>482.4238, 563.0436, 757.5022<br>906.1623, 1093.7616, 1128.3767<br>1378.1142, 1403.4592, 1454.4251<br>1469.3939, 1653.0489, 2950.6366<br>3005.5726, 3067.2315, 3130.2605

Stationary Point	Cartesian Coordinates	Vibrational Frequencies (cm^{-1})
**TS10**	C -1.22203 -0.24067 -0.00799<br>C -0.02721 0.33780 0.19406<br>H -1.30331 -1.21240 -0.47238<br>H -2.12266 0.29242 0.25090<br>H -0.08761 1.54533 -1.35970<br>H 0.06590 1.27096 0.73548<br>O 1.13244 -0.34152 -0.03868<br>H 1.88362 0.25308 0.03867	617.9098*i*, 299.0658, 392.6144<br>410.3720, 489.1787, 683.3090<br>847.5904, 956.2535, 995.2287<br>1141.9787, 1274.5297, 1330.9794<br>1439.7846, 1639.1730, 3156.6244<br>3166.6939, 3262.1224, 3833.3455
**TS11**	C -1.25448 -0.21788 0.02843<br>C 0.04274 0.47576 -0.04281<br>H -1.26081 -1.28182 -0.14834<br>H -2.09346 0.24986 0.52123<br>H -0.78148 0.38640 -1.04774<br>H 0.09425 1.48954 0.34119<br>O 1.17272 -0.31722 0.08785<br>H 1.93014 0.14646 -0.28288	1914.3803*i*, 351.2801, 402.6078<br>440.3918, 713.6469, 790.7011<br>943.1311, 1083.7479, 1166.0980<br>1253.9541, 1298.2404, 1397.2775<br>1440.7885, 2138.3085, 3108.5101<br>3144.7056, 3262.9565, 3808.5926
**TS12**	C -1.31068 -0.21862 0.00001<br>H -1.73599 -0.59330 0.93825<br>H -1.73592 -0.59347 -0.93821<br>O -0.32093 0.54695 -0.00003<br>C 1.48722 -0.18776 0.00001<br>H 2.02541 0.74647 -0.00020<br>H 1.47741 -0.74833 0.92226<br>H 1.47731 -0.74869 -0.92201	549.9285*i*, 86.3363, 159.7900<br>285.7049, 645.9498, 673.6656<br>938.6733, 980.6253, 1217.1380<br>1349.5298, 1427.1683, 1431.9780<br>1553.8828, 2965.7741, 3045.3422<br>3103.1554, 3260.9976, 3271.0037
**TS13**	C 0.81548 -0.46496 -0.00000<br>H 1.22737 -0.85639 0.93018<br>H 1.22737 -0.85639 -0.93018<br>O 0.36073 0.82395 0.00000<br>C -1.02490 -0.20431 0.00000<br>H -1.21406 -0.77663 -0.90425<br>H -1.65594 0.69001 -0.00004<br>H -1.21406 -0.77656 0.90429	1225.1081*i*, 164.3285, 477.7913<br>521.0132, 899.5561, 981.8615<br>1042.3710, 1152.5187, 1208.6418<br>1345.0989, 1435.6834, 1472.0213<br>1485.3998, 2969.2984, 3010.1524<br>3062.2864, 3116.8128, 3147.9164
**TS14**	C -0.87818 -0.30655 0.00000<br>H -1.39816 -0.51755 0.92862<br>H -1.39816 -0.51755 -0.92862<br>O -0.05303 0.81680 0.00000<br>C 0.72857 -0.38791 0.00000<br>H 2.06129 0.33900 0.00000<br>H 1.02843 -0.83578 0.94092<br>H 1.02843 -0.83578 -0.94092	1313.7778*i*, 472.0280, 538.0657<br>715.9609, 97.5458, 948.1717<br>1040.6885, 1067.3416, 1154.5833<br>1163.3850, 1217.7623, 1224.1242<br>1444.6133, 1501.3155, 3053.7407<br>3071.8135, 3173.1364, 3186.5497
**TS15**	C 1.15159 -0.29712 -0.03951<br>C 0.16984 0.67268 0.22711<br>H -0.24621 -0.82113 -0.45588<br>H 2.02198 -0.01503 -0.62294<br>H 0.05715 1.60270 -0.31292<br>O -1.18711 -0.18563 -0.14054<br>H -1.61905 -0.47755 0.67640<br>H 1.35444 -1.05728 0.71406	1732.4938*i*, 351.2462, 438.8974<br>556.4474, 746.5385, 756.2952<br>819.5551, 904.6088, 1055.9069<br>1181.6499, 1276.0769, 1369.8955<br>1470.0985, 1658.3932, 3059.3936<br>3143.3268, 3185.5854, 3726.3369

Stationary Point	Cartesian Coordinates	Vibrational Frequencies (cm^{-1})
**TS16**	C -1.25410 -0.27282 -0.00254<br>C -0.00428 0.51865 -0.03532<br>H -1.23269 -1.32783 -0.23016<br>H -2.20138 0.20918 0.19228<br>H 0.07938 1.08589 -0.97541<br>H -0.01198 1.27075 0.76274<br>O 1.15498 -0.30239 0.14248<br>H 1.67714 -0.29383 -0.66212	254.4967*i*, 114.7130, 419.9643<br>472.7117, 845.3355, 933.0778<br>1076.4714, 1098.0140, 1179.5576<br>1339.2185, 1395.4624, 1447.0272<br>1474.4690, 2920.4448, 2984.1385<br>3148.0102, 3256.3224, 3854.2860
**TS17**	C 1.22623 -0.18985 0.00106<br>C -0.07853 0.50737 -0.08943<br>H 1.28556 -1.02573 -0.70238<br>H 2.04483 0.49841 -0.21015<br>H -0.17115 1.56778 0.11773<br>O -1.22273 -0.23372 0.12799<br>H -1.65415 -0.46425 -0.70163<br>H 1.39051 -0.61161 1.00270	455.7373*i*, 171.5682, 400.7524<br>469.7024, 910.6624, 985.9714<br>1107.8693, 1126.1256, 1234.5928<br>1370.8901, 1411.3638, 1462.0894<br>1479.1312, 2956.6022, 3022.1906<br>3090.9572, 3139.0360, 3770.0053
***cyc*-$CH_2(O)CH_2$**	C 0.00000 0.73173 -0.37316<br>O 0.00000 0.00000 0.85471<br>C 0.00000 -0.73173 -0.37316<br>H 0.91790 1.26693 -0.58996<br>H -0.91790 1.26693 -0.58996<br>H -0.91790 -1.26693 -0.58996<br>H 0.91790 -1.26693 -0.58996	819.8890, 843.6593, 887.7158<br>1047.8239, 1149.3074, 1167.4407<br>1169.2798, 1176.5335, 1298.7475<br>1505.2778, 1538.2550, 3086.3881<br>3092.5634, 3165.4513, 3180.2921
**H_2O**	O 0.00000 0.00000 0.11700<br>H 0.00000 0.76354 -0.46798<br>H 0.00000 -0.76354 -0.46798	1627.0047, 3795.9910, 3898.7171
**CH_3**	C 0.00000 0.00000 0.00046<br>H 0.00000 1.07813 -0.00091<br>H -0.93369 -0.53907 -0.00091<br>H 0.93369 -0.53907 -0.00091	542.5949, 1407.6998, 1407.7001<br>3108.1578, 3282.9149, 3282.9156
**H_2CO**	C 0.00000 0.00000 -0.52730<br>O 0.00000 0.00000 0.67317<br>H 0.00000 0.93871 -1.11080<br>H 0.00000 -0.93871 -1.11080	1196.6460, 1262.9303, 1529.2060<br>1811.7531, 2889.5734, 2945.8221
**CH_2CH**	C 0.04804 -0.58430 0.00000<br>C 0.04804 0.71689 0.00000<br>H -0.88019 -1.15847 0.00000<br>H 0.96502 -1.16520 0.00000<br>H -0.66126 1.52812 0.00000	707.1637, 820.4781, 928.3537<br>1047.1243, 1395.2504, 1650.2285<br>3041.0167, 3137.8355, 3241.3599

Stationary Point	Cartesian Coordinates	Vibrational Frequencies (cm^{-1})
***anti*-CH_2CHOH**	C 1.22354 -0.17995 0.00003<br>C 0.03847 0.41265 -0.00000<br>H 1.31820 -1.25644 0.00007<br>H 2.12027 0.41913 -0.00004<br>O -1.13289 -0.29479 -0.00007<br>H -0.06696 1.49179 -0.00012<br>H -1.88047 0.30759 0.00049	281.3302, 484.5160, 714.9233<br>867.1391, 56.9790, 976.5655<br>1138.6335, 1280.6670, 1348.9047<br>1441.4237, 1721.8901, 3146.8985<br>3164.1468, 3253.0070, 3848.7091
***syn*-CH_2CHOH**	C -1.19831 -0.20685 -0.00000<br>C -0.03907 0.43983 0.00000<br>H -1.25735 -1.28818 -0.00000<br>H -2.12355 0.34745 0.00000<br>O 1.20544 -0.11280 -0.00000<br>H 0.02691 1.51951 0.00000<br>H 1.13475 -1.07429 0.00001	456.2998, 491.8697, 713.0194<br>842.6968, 960.1810, 1000.3379<br>1116.5011, 1323.4792, 1349.8371<br>1450.6308, 1695.5666, 3143.7039<br>3188.5054, 3241.2843, 3787.8554
**CH_3CHO**	C -1.16563 -0.14847 0.00003<br>C 0.23247 0.39617 -0.00000<br>H -1.70155 0.22625 0.87601<br>H -1.16174 -1.23579 0.00016<br>H -1.70123 0.22584 -0.87638<br>O 1.23213 -0.27577 0.00001<br>H 0.30648 1.50368 -0.00002	157.9592, 509.4678, 774.6956<br>886.0493, 1129.1653, 1135.7970<br>1378.8274, 1422.3081, 1459.9533<br>1469.1778, 1806.0560, 2868.9566<br>3022.4072, 3073.3247, 3134.8917

Equilibrium concentration ratio between the *syn-/anti*-CH_2CHOH conformers

The equilibrium constant for the *anti*-CH_2CHOH ⇄ *syn*-CH_2CHOH reaction was calculated considering the partition function of the two species:

$$\frac{q_{syn}}{q_{anti}} \times exp^{\frac{-\Delta E}{kT}}$$

where ΔE is the difference in the ground state energy ($E_{0,syn}$ - $E_{0,anti}$).

The global partition function was obtained considering both the rotational (q_{rot}) and the vibrational (q_{vib}) partition functions:

$$q = q_{rot} \times q_{vib}$$

The rotational partition function is expressed as:

$$q_{rot} = \frac{\sqrt{\pi}}{\sigma}\sqrt{\frac{T^3}{\Theta_A \Theta_B \Theta_C}}$$

which can be simplified as follows:

$$q_{rot} = \sqrt{\frac{1}{\Theta_A \Theta_B \Theta_C}}$$

The rotational temperature Θ_r [K] is proportional to the rotational constant B [Hz]:

$$\Theta = \frac{Bh}{k}$$

where *h* is the Plank's constant and *k* is the Boltzmann constant. Similarly, the vibrational partition function is expressed considering the vibrational temperatures Θ_v :

$$\Theta_v = \frac{vch}{k}$$

$$q_{vib} = \prod_{i=1}^{n} \frac{1}{1-e^{-\frac{\Theta_v}{T}}}$$

where *c* represents the speed of light, *n* is the number of vibrational modes and ν is the frequency (in cm^{-1}). The values of the rotational and vibrational temperatures (Θ_r and Θ_v respectively) were obtained through *ab initio* calculations on the two *anti*-CH_2CHOH and *syn*-CH_2CHOH conformers.

References

(1) Senosiain, J. P. ; Klippenstein, S. J.; Miller, J. A. Reaction of Ethylene with Hydroxyl Radicals: a Theoretical Study. *J. Phys. Chem. A* **2006**, *110*, 6960–6970.

(2) Zhu, R.; Park, J.; Lin, M. C. Ab Initio Kinetic Study on the Low-Energy Paths of the HO + C_2H_4 Reaction. *Chem. Phys. Lett.* **2005**, *408*, 25–30.

(3) Zhang, Y.; Zhang, S.; Li, Q. S. Ab Initio Calculations and Mechanism of Two Proton Migration Reactions of Ethoxy Radical. *Chem. Phys.* **2005**, *308*, 109–116.

(4) Liu, G.-X.; Ding, Y.-H.; Li, Z. S.; Fu, Q.; Huang, X. R.; Sun, C.-C.; Tang, A.-C.; Theoretical Study on Mechanisms of the High-Temperature Reactions C_2H_3 + H_2O and C_2H_4 + OH. *Phys. Chem. Chem. Phys.* **2002**, *4*, 1021–1027.

(5) Alvarez-Idaboy, J. R.; Mora-Diez, N.; Vivier-Bunge, A. A Quantum Chemical and Classical Transition State Theory Explanation of Negative Activation Energies in OH Addition to Substituted Ethenes, *J. Am. Chem. Soc.* **2000**, *122*, 3715–3720.
